# A High-Throughput Experimental Approach to Screening Gas Sorption by Liquids and Solids

**DOI:** 10.1021/acssuschemeng.3c05901

**Published:** 2023-12-07

**Authors:** J. Mark Young, Sam H. McCalmont, Sophie Fourmentin, Panagiotis Manesiotis, John D. Holbrey, Leila Moura

**Affiliations:** † QUILL Research Centre, Queen’s University Belfast, School of Chemistry and Chemical Engineering, David Keir Building, 39-123 Stranmillis Road, Belfast, BT9 5AG, United Kingdom; ‡ Unité de Chimie Environnementale et Interactions sur le Vivant (UCEIV), EA 4492, Condorcet FR CNRS 3417, Université du Littoral-Côte d’Opale, 59140 Dunkerque, France; § Queen’s University Belfast, School of Chemistry and Chemical Engineering, David Keir Building, 39-123 Stranmillis Road, Belfast, BT9 5AG, United Kingdom

**Keywords:** Gas uptake, Gas solubility, Gas separation, Gas capture, High-throughput screening, CO_2_, CH_4_, Ethylene

## Abstract

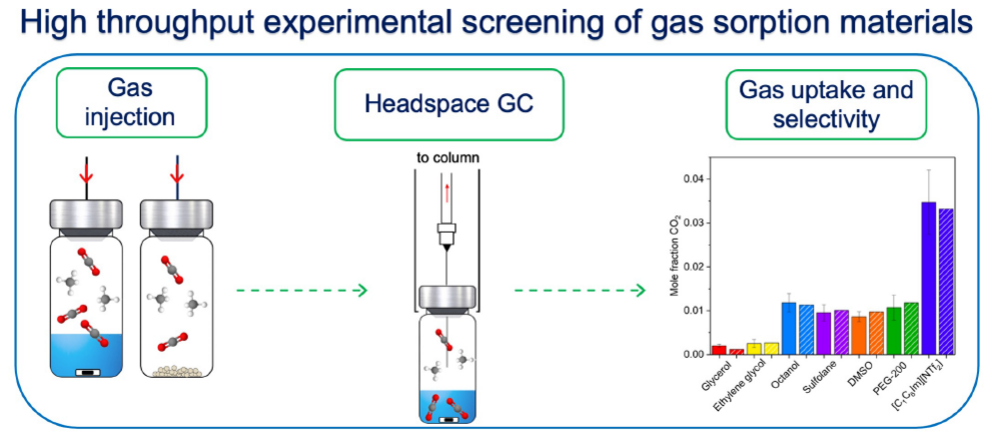

High-precision measurement of gas uptake from single or mixed feeds in solid and liquid sorbents traditionally requires time-consuming experimental procedures and/or complex and costly equipment. A simple and cost-effective headspace gas chromatography (HS-GC) approach for the fast, universal experimental screening of sorbents for gas uptake and/or determination of their real gas separation selectivity has been developed and is demonstrated for pressures up to 2500 mbar and temperatures above 30 °C. This method allows screening of solids and both volatile and nonvolatile liquid materials, physisorbents, and chemisorbents using both single and mixed permanent gases that can include CO_2_, CH_4_, H_2_, and NH_3_, for gas uptakes as low as 0.04 mmol or 1.8 mg of CO_2_. We estimate that this method allows for the screening of at least 30–96 sorbents (in triplicate) or 90–264 sorbents (singles) per day, representing at least a 90–3000 times reduction in the time required for equivalent analysis.

## Abstract 2

This method enables, for the fist time, the screening of a large variety and number of samples for gas capture and separation applications.

## Introduction

Gas capture and separation research holds significant importance due to its contribution to the development of technologies related to energy security and efficiency, climate change mitigation, and the integration of renewable energies. To do this, knowledge of multicomponent phase equilibria is crucial to design separation processes and equipment, perform catalytic reactions, and effective gas capture and storage. The current interest in developing CO_2_, CH_4_, H_2_, and NH_3_ capture, transport, conversion, and/or storage applications using materials such as microporous solids, ionic liquids (ILs), deep eutectic solvents (DES), and porous liquids (PLs) has led to a steady increase in publications on gas–liquid and solid phase equilibria.^1−5^ However, progress is limited by the lack of experimental screening methods that can assess a large number of samples quickly.

A variety of methods have been developed to address the challenges associated with the measurement of vapor–liquid equilibria (VLE).^6^ The most popular of these methods are either synthetic, where the properties of a precisely known mixture are investigated or analytical, where compositions of equilibrium phases are determined.^6^

Examples of synthetic methods include volumetric and bubble point analyses (for liquids). Volumetric techniques measure the volume, or associated pressure drop, of sorption of a gas by a known amount of sorbent.^7^ The bubble point technique determined the temperature/pressure points at which bubbles initially form from a gas saturated liquid.^8^ Such techniques require relatively simple equipment and have potential for miniaturization; however, the precision required for the preparation of the mixtures and the determination of additional properties required for sorption calculations (e.g., phase densities) can be challenging. Additionally, there is less information that can be directly obtained from these measurements in comparison with analytical techniques, and so synthetic methods are most useful for binary systems.^6^

Analytical methods include gravimetric (microbalance or bubbling/weighing), NMR and (inverse) gas chromatography.^9^ Gravimetric methods are commonly applied to solids and low vapor pressure liquids, the simplest approach being to weigh the sorbent before and after contact with gas samples.^10^ Gravimetric microbalances require only very small sample sizes to make accurate measurement of solubility and diffusivity simultaneously for absorption and desorption isotherms.^7,11,12^ NMR has also been used to determine gas uptake by pressurizing the sorbent with the desired gas in a high-pressure NMR tube. Although affording less accurate results, an advantage of this technique is that additional structural or reaction information can be obtained.^13^ Gas and gas–liquid chromatography (GC and GLC) can be used to determine infinite dilution activity coefficients of volatile liquids or gases in low volatility liquid sorbents coated on a stationary gas chromatography phase.^14^ Retention times are correlated with the infinite dilution activity coefficients of the solutes and solubilities in the form of Henry’s Law constants and thermodynamic properties of solvation have been derived.^15,16^ Typical difficulties associated with analytical methods are related to the accurate determination of the composition of the phases in equilibrium, for example, the need to add calibration data or account for buoyancy effects.

The majority of studies in the relevant literature are devoted to single gas uptake and assume ideal mixing and selectivities for the mixed gas separation. While this is a good approximation for ideal systems or when the solubility/uptake of the gas is low, for complex, high capacity, or reactive systems, experimental data for mixed gas separation becomes more critical, particularly in view of commercial applications.

Combined methods have been developed to study multicomponent gas mixtures, for example, using a volumetric or gravimetric technique associated with a single qualitative GC analysis of the headspace. This still involves, however, the *in situ* measurement of one system at the time after equilibrium is reached and generates the disturbance of said equilibrium. Naturally it then requires longer or a larger number of measurements.^17,18^

Here we describe a new method that combines the pressure drop technique on samples contained in pressurized headspace GC (HS-GC) vials (between 500 and 2500 mbar) with quantitative GC analysis of the equilibrated headspace.

Simple sample preparation and loading, simultaneous equilibration of a large number of samples, and straightforward quantitative headspace gas analysis allows rapid streamlining compared to typical solubility or gas-uptake measurements by both solid and liquids sorbents across a range of moderate temperatures and pressures, with either pure gases or gas mixtures.

The universality of this methodology has been demonstrated by benchmarking with single and mixed CO_2_, CH_4_, C_2_H_6_, and C_2_H_4_ gases and a variety of solid (molecular sieves 3 and 4 Å, and zeolite RHO) and liquid sorbents including organic solvents (glycerol, 1-octanol, dimethyl sulfoxide, sulfolane, ethylene glycol, and polyethylene glycol 200), aqueous monoethanolamine, ionic liquids with and without added silver salts, and a type-III porous liquid (zeolite RHO in Genosorb).

To our knowledge, this is the first fast experimental screening method available for determining gas uptake/solubility and real separation selectivity and for such a variety of sorbents and without disturbing or manipulating the sorbent.

## Experimental Section

### Materials

Dimethyl sulfoxide (DMSO, purity 99.9%), polyethylene glycol 200 (PEG200), glycerol (anhydrous for synthesis), ethylene glycol (purity 99%), 1-octanol (purity 99%), and sulfolane (purity 99%) were used as purchased from Sigma-Aldrich. Molecular sieves types 3 and 4 Å were purchased from Thermo scientific and were activated in a vacuum oven overnight at 150 °C. Zeolite RHO and zeolite RHO in Genosorb 1753 at 25 wt % (porous liquid) were kindly provided by Porous Liquid Technologies. Zeolite RHO was activated overnight at 200 °C under vacuum. 1-Methylimidazole (purity of 99%) was purchased from Doug Discovery, 1-chlorobutane (purity of 99.5%), silver nitrate (purity of 99%), and sodium hydroxide (purity of 97%) were purchased from Sigma-Aldrich and lithium bis(trifluoromethanesulfonyl)imide (Li[NTf_2_]) was purchased from 3M (noted as “highly pure”). The aqueous monoethanolamine (MEA) solution was made by mixing deionized water with MEA at a concentration of 30 wt % in MEA. Ionic liquids, 1-butyl-3-methylimidazolium chloride ([C_4_C_1_Im]Cl), bis(trifluoromethanesulfonyl)imide ([C_4_C_1_Im][NTf_2_]), and 1-hexyl-3-methylimidazolium bis(trifluoromethanesulfonyl)imide ([C_6_C_1_Im][NTf_2_]), were prepared in-house as described in Supporting Information. The silver salt mixture with [C_4_C_1_Im][NTf_2_] contained 0.25 mol L^–1^ of Ag[NTf_2_]. The water content of the sorbent materials (when relevant) is reported in Supporting Information. Carbon dioxide (CO_2_), methane (CH_4_), ethane (C_2_H_6_), ethylene (C_2_H_4_), and 1:1 molar mixtures of CO_2_/CH_4_, and C_2_H_6_/C_2_H_4_ (all 99.95% purity) were supplied by BOC and used as received.

### Headspace Gas Analysis

Gas analysis was carried out using a PerkinElmer Clarus 500 gas chromatograph (GC) attached to a Turbomatrix 40 headspace (HS) autosampler using helium as a carrier and a flame ionization detector (FID) equipped with a methanizer using a nickel catalyst. The HS autosampler oven was set to equilibrate samples for 2h at 35 °C (308 K) as the lowest possible temperature for this particular piece of equipment, but equilibration temperature can be easily modified. The method included a high pressure injection mode with a 40 psi injection for 2 min and a column pressure of 15 psi withdraw time of sample is 6 s withdraw time before the vial was vented. The needle transfer and line temperatures were set to 50 °C. The GC injector temperature was set to 50 °C, the oven to 70 °C and the FID detector and methanizer set to 350 °C with an H_2_ flow of 45 mL/min. The carrier gas (helium) pressure within the column was set to 12.5 psig and has a split flow of 10 mL/min. In all experiments the GC column used was an Agilent J&W CP-Silica PLOT with a nominal geometry of 15 m × 0.32 μm i.d. × 4 μm d.f. Characteristic chromatographs with retention times for all gases are included in the Supporting Information. PerkinElmer 20 mL crimp CTC headspace vials (rated to 5.17 bar) and gray butyl stoppers (part number B0038137) were used for all experiments due to their superior sealing capabilities, including resealing after being pierced with 23S or 20 gauge point style 2 Hamilton luer lock needles and maintaining pressures up to 2.5 bar over 2 weeks.

### Method Description

**Figure 1:**
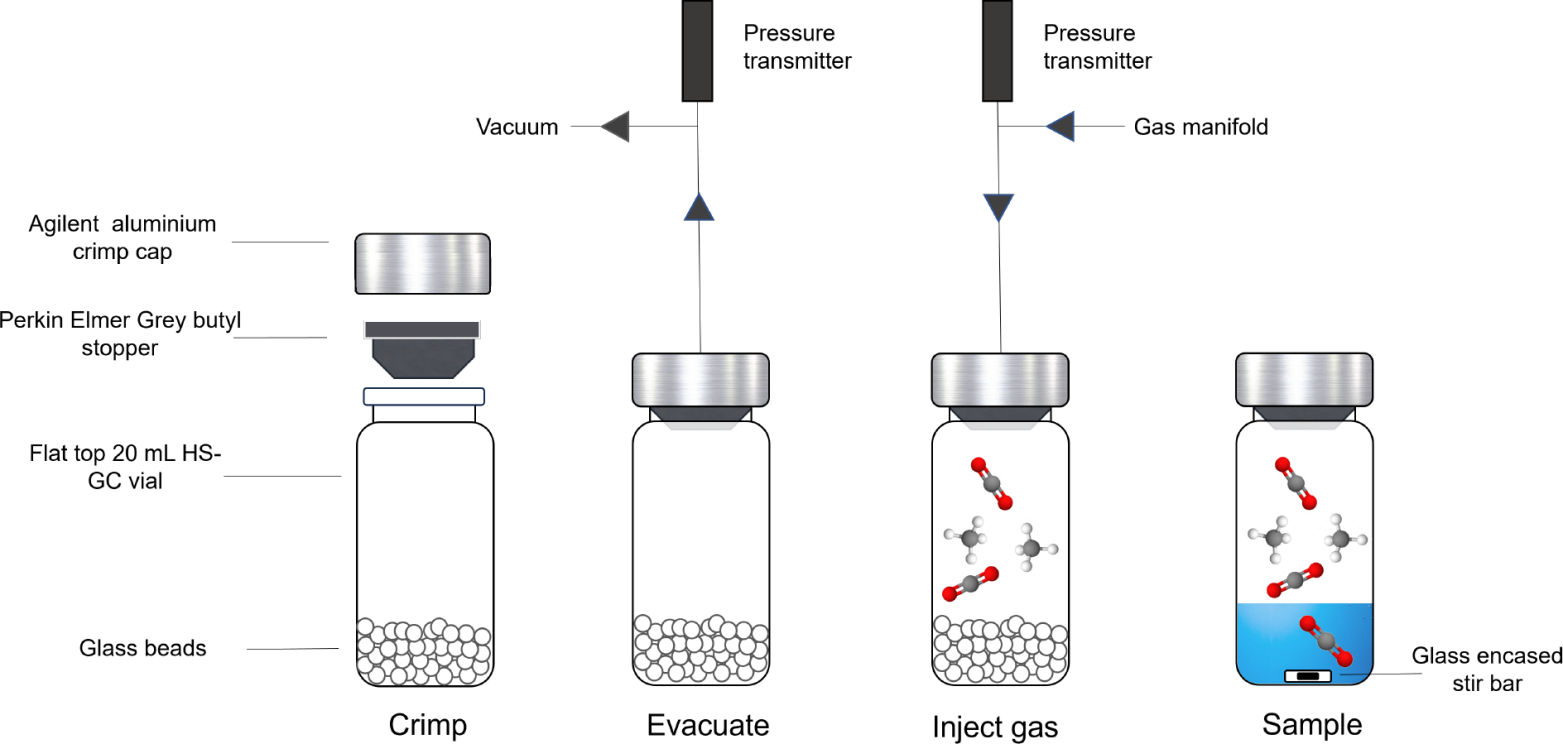
Summary of the sample preparation process for vials with glass beads or with liquid sorbents in headspace gas chromatography (HS-GC) for calibration curves or gas uptake experiments, respectively. This includes the method for measuring the pressure in the vial with a pressure transmitter.

The first step of the method was to produce a calibration curve corresponding to a linear response of the detector to a range of pressures in the samples vials. To do this, a series of closed HS-GC vials at a variety of gas pressures were produced, in triplicate, either empty or containing predetermined volumes of glass beads (see Figure 1). The occupied volume (if any) in the calibration vials was set to ensure consistency in the results for sorbent samples occupying a large portion of the space. The glass beads served this purpose when needed, as a nonsorbing material. The HS-GC response to each gas was measured between pressures ranging from approximately 500 to 4000 mbar at 308 K.

Then, HS-GC vials containing a similar volume of sorbent samples as the calibration curve were produced and pressurized with the same gases at the same range of pressures. Air or water sensitive samples can be easily prepared in a glove box or bag under a controlled atmosphere and used with this method. Samples can be speedily produced, in triplicate and in large quantities, and left to equilibrate *ex situ*, affording this method another one of its advantages over traditional techniques. After equilibration, the detector response to the headspace gas samples can be correlated with the gas’ pressure in the vial’s free volume, and the gas uptake by the sorbent can be thus calculated.

**Figure 2:**
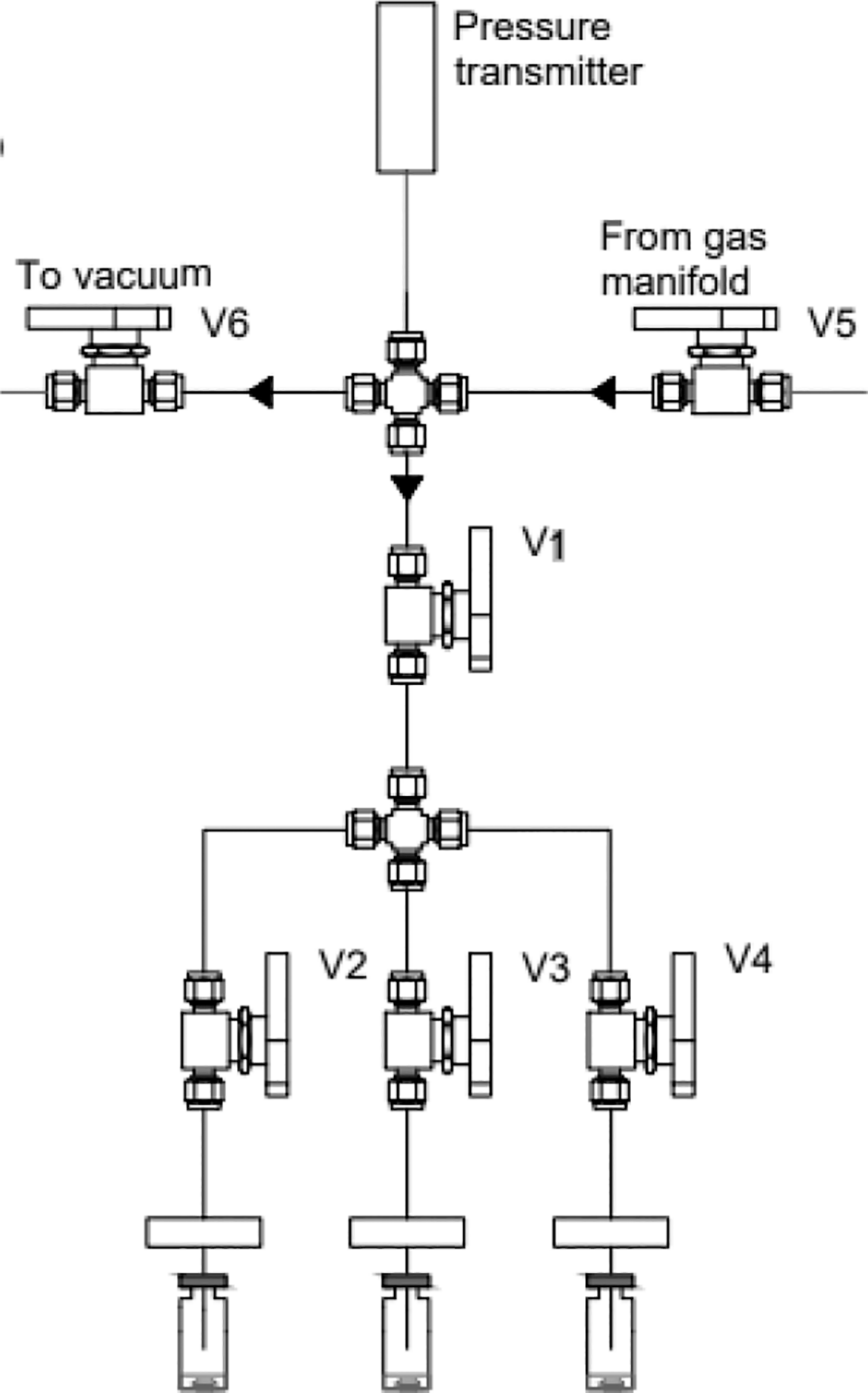
Schematic of the “trident” stainless-steel gas system setup used to produce three identical samples simultaneously, at the same initial gas pressure. V1–V6 represent Swagelok ball valves. The needles are represented piercing three sealed headspace gas chromatograph vials through gray butyl stoppers.

The method for sample or calibration vial preparation can be divided in 3 steps: pressurization, equilibration, and HS-GC measurement. The pressurization process is summarized in Figure 1. The vials’ headspace is first evacuated of any gases by applying vacuum through a needle piercing the gray septa and connected to a gas/vacuum line (see Figure 2). Then the vial is pressurized with the gas(es) and pressures of choice, which is accurately measured. The vials are then removed from the gas line and left to equilibrate *ex situ* at the required temperature. After equilibration, the HS-GC response measurement can be performed.

The sample amount/volume was optimized for the system of choice beforehand. This means that the amount of sorbent should capture a sufficiently large amount of gas from the headspace in comparison with the calibration curve at the same initial pressure, to produce reliable results above the uncertainty level of each data set. The optimal amount of sorbent was quickly determined by testing a range of quantities. For the systems chosen here, sample amounts between 3 and 10 mL for liquids were used. For example, for CO_2_ experiments with liquid physisorbents, approximately 4.8 mL of sample was used, while 9.8 mL was necessary for CH_4_ measurements. This difference is due to the large difference in solubility in the absorbents tested (see Figure 4 and Supporting Information for comparison). The porous solid sorbents used presented substantially higher gas uptake capacities per mass than the liquids; therefore, only approximately 0.5 g of solid was needed. A glass encased stir bar of approximately 0.2 mL was included in all liquid samples to promote faster equilibration times.

The quantity of gas sorbed is calculated using eq 1 from the difference between a pressure–volume–temperature (*pVT*) measurement and the GC peak area measurement. First, the amount of gas introduced in the vial, *n*_*g*_^tot^, is determined from the measurement of its pressure and estimated room temperature (initial pressure and temperature, *p*_ini_ and *T*_ini_, respectively), in the predetermined vial volume, *V*_vial_. This volume is obtained by measurement of the weight of distilled water filling a model vial, minus the volume of sorbent (if liquid), the volume of the glass encased stir bar, or glass beads present. For solid sorbents, the occupying volume was considered negligible.
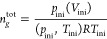
1Deviations from ideal behavior for the gases were considered by the addition of compressibility factor to eq 1, however, the corrections were much smaller than the uncertainty associated with the measurements, even at the highest pressures, and so were neglected.

The GC sampling and measurement is performed after thermodynamic equilibrium is reached between the gas and the known quantity of predegassed sorbent. The amount of gas remaining in the headspace of the vial, *n*_g_^HS^, is calculated as
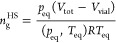
2where *p*_eq_ and *T*_eq_ are the pressure and temperature at equilibrium. We considered that the volume of the liquid phase does not change after gas uptake and that the contribution to the partial pressure from the liquid sorbents was negligible at the temperatures considered. This means that the amount of sorbent, *n*_sorb_, remains constant. These approximations are estimated to have a negligible effect on the accuracy of the gas absorption measurements within our estimated uncertainty levels.

The pressure at equilibrium is estimated from the corresponding GC peak area, using the instrument response calibration curve as reference. The amount of gaseous solute sorbed by the sample, *n*_g_^sorb^, can then be determined from
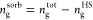
3For mixed gas solubility measurements, all pressures expressed correspond to partial pressures of the specified gas. In these cases, the equilibrium pressure, *p*_eq_, is determined by taking into account the total pressure obtained via HS-GC results and the molar proportion of the gas from the peak area fraction.

Gas solubility was expressed as the solute mole fraction (*x*_*gas*_) or sorbent capacity (*C*_*gas*_), calculated from the amount of gaseous solute absorbed by the sorbent, from eqs 4 and 5, respectively:
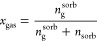
4The sorbent capacity is given by eq 5.
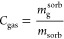
5where *m*_g_^sorb^ is the mass of gas sorbed by the sorbent and *m*_sorb_ is the mass of sorbent.

For two gases (designated 1 and 2) the ideal gas separation selectivity (α_i_) of a sorbent is given by the ratio of their respective solubilities, as exemplified in eq 6 for mole fraction solubilities
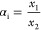
6or by the same mol/mass fraction ratio at the same equilibrium partial pressure and temperature. Real gas separation selectivity α_r_ of the sorbents is directly determined from the gases’ relative solubility (here in mole fraction) in each sorbent via HS-GC mixed gas experiments, performed as described above and with a corresponding mixed gas calibration curve.

### Sample Pressurization

HS-GC vials were loaded with a known amount of sorbent or glass beads, sealed with an autocrimper using a stopper and an aluminum crimp cap, placed on the “trident” gas system described in Figure 2, and purged under vacuum through the system’s needles for either 3 or 15 min depending on the internal diameter of the needle used. A trio of closed vials can be placed in the trident and pressurized with the chosen gas/gas mixture simultaneously. This produces 3 vials at the same pressure, and a triplicate measurement.

The stainless-steel “trident” gas system was constructed to expedite sample preparation and is shown schematically in Figure 2. It consists of three male Luer lock fittings to 1/4 in. to 18 in. NPT thread fitting adapters (TSD931–1418MSS) supplied by Adhesive Dispensing. These were welded to a Swagelok tube fitting female connector, 1/4 in. tube OD × 1/4 in. female NPT to form a gastight seal. To each of the luer lock side a 10.5 mm × 20 G or a 51 mm × 23S G male luer lock needle in stainless steel with point style 2 was added, leaving a gastight seal. The internal diameter of the injection needles was selected to maximize the rate of gas flow in and out of the vials to minimize the time required to obtain the “initial pressure” measurement, *p*_ini_ (see eq 1), without generating leaks by compromising the integrity of the pierced septa. The 1/4 in. connection from the Swagelok adaptor was then fitted to the gas system via 1/4 in. pipe. Each trident head has its own ball valve (Swagelok SS-42GS4). The system is connected to a vacuum pump and gas regulators and the corresponding cylinders.

The pressure of the system was monitored by a Keller Pressure Series 33X pressure transmitter, operating between 0 and 6 bar absolute and with a precision of 0.001 bar. The bulk of the remainder of the system is built with Swagelok parts. This includes pipework (SS-T4-S-035–6ME, 1/4 in. OD × 0.035 in. wall thickness) and ball valves (SS-42GS4).

## Results and Discussion

In the first instance, we produced calibration curves for CO_2_, CH_4_, C_2_H_4_, and C_2_H_6_ and two of their mixtures, CO_2_/CH_4_ and C_2_H_4_/C_2_H_6_ (see data in Supporting Information). These correlated the pressure in the HS-GC vials to an integrated GC peak area. The calibrations were obtained for a variety of free vial volumes, between empty vials and vials occupied with 10 mL of glass beads, to match the volumes occupied by different types of sorbent samples. The HS-GC response to each gas was measured between pressures ranging from approximately 500 mbar to 4000 mbar at 308 K. A linear response range was obtained for pressures up to 2500 mbar and this correlation was used for the solubility measurements. A wide variety of gases (and gas mixtures) and sorbent materials was tested, from liquids to solids, physisorbents to chemisorbents, to demonstrate the versatility of the method.

### Detector Response and Calibration

**Figure 3:**
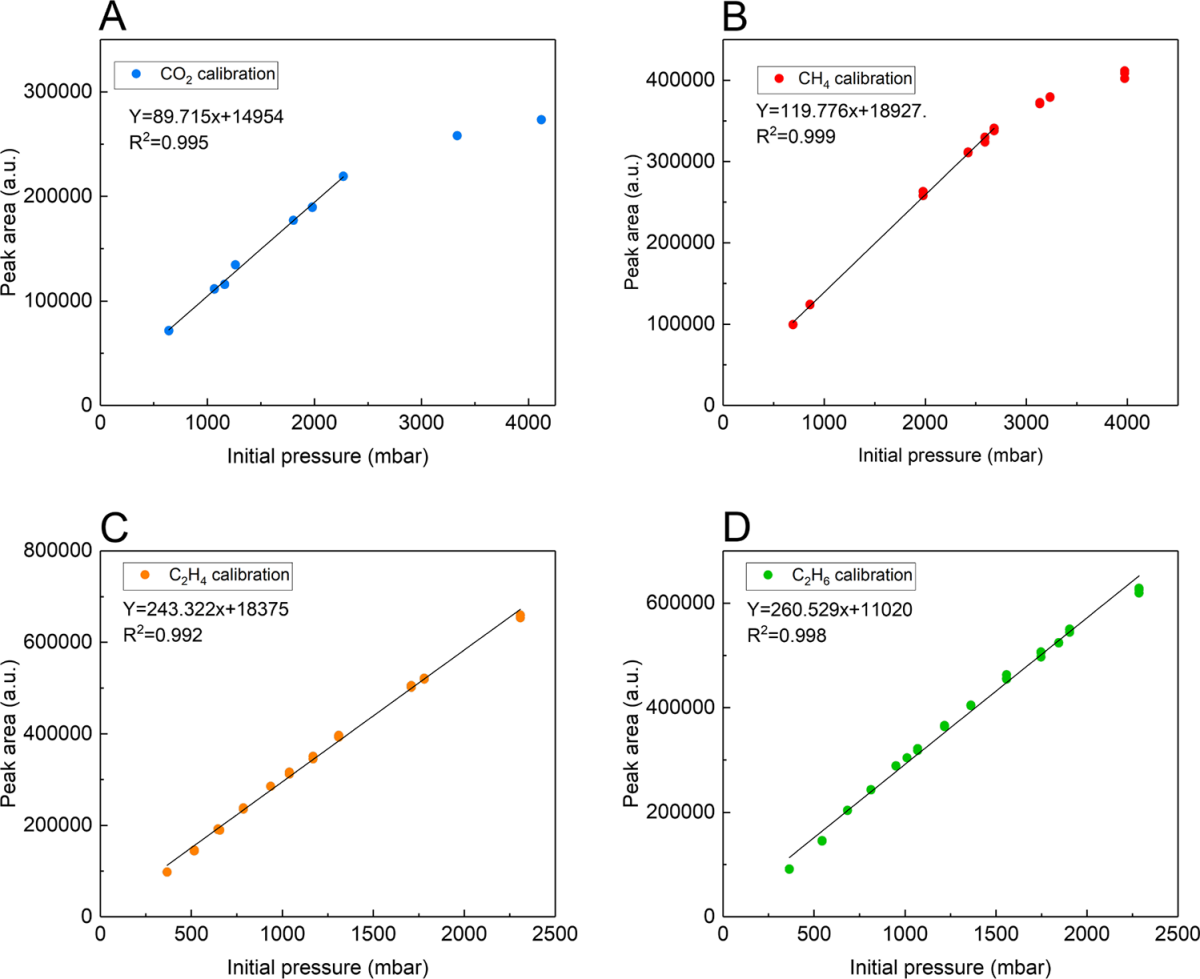
Response calibration profile for GC peak area to changes in initial gas partial pressure, *p*_ini_, between 500–4000 mbar at 308 K for carbon dioxide (A, blue symbols, top left), methane (B, red symbols,top right), (C, orange symbols, bottom left), and ethane (D, green symbols, bottom right), with with HS-GC vial occupied volume by glass beads of 5, 5, 3, and 3 mL, respectively. All data points were obtained in triplicate.

Empty vials or vials containing glass beads were filled with pure gas or a mixture of gases at a range of pressures between 500 and 4000 mbar, and their corresponding peak areas were measured via HS-GC. Each measurement was conducted in triplicate by loading three equivalent but independent vials on the “trident” gas system. Some of the GC integrated peak areas as a function of the initial gas pressure applied to the vials for CO_2_, CH_4_, C_2_H_4_, and C_2_H_6_ gases are shown in Figure 3. All raw data can be found in the Supporting Information. Reasonable linearity was obtained for all gases and glass bead volumes up to a pressure of 2500 mbar, with *R*^2^ values of greater than 0.99. Linear correlation fits, correlation coefficients (*R*^2^) and standard deviations for each system along with measurement repeatability assessments are reported in the Supporting Information. This upper pressure range restriction is likely due to equipment limitations and could be extended if needed with different equipment and/or changes of the HS-GC method parameters.

We observed that the calibration curves for the same gas in vials with different free volumes presented small but consistent peak area differences. For example there was a 14% difference in the CO_2_ calibration peak area when using 10 mL of glass beads when compared to using empty vials. For this reason, we have measured a range of calibration curves and use the closest one to the volume of sorbent for the required calculations. From these results we can also establish that small variations in liquid sample volume versus bead volume do lead to peak area variations, but we estimated these to be within the method’s uncertainty. For solid sorbents, the sample volumes were so small that the corresponding calibration curves used for the calculations were measured by using empty HS-GC vials.

### Gas Uptake for Pure Gases

Gas uptake into a series of sorbents was examined using the screening method developed, combining pressurization of gas over sorbents in HS-GC vials for pressures from 500–2500 mbar, and at 308 K, with direct sampling and gas analysis to determine partial pressures and compositions of the remaining, nonsorbed, gas.

A range of industrially and environmentally relevant permanent gases, CO_2_, CH_4_, C_2_H_4_, and C_2_H_6_ have been examined, in addition to two 1:1 mol gas mixtures, CO_2_/CH_4_ and C_2_H_4_/C_2_H_6_, to illustrate the potential to evaluate real gas separation selectivity α_r_. Gas sorption was screened using both solid and liquid sorbents that can take up specific gases through either chemsorption or physisorption mechanisms, demonstrating the broad applicability of the methodology developed.

For materials with fast sorption kinetics such as porous solid sorbents and the aqueous MEA solution, the 20 G needle was used. In these cases 3 min of vacuum was applied. With volatile liquids, for example, aqueous MEA, the liquid was frozen using liquid nitrogen prior to evacuation and the weight of the sample after evacuation checked to ensure no significant sorbent loss. The contribution of the liquid to the final partial pressure of the headspace was estimated to be not significant within our estimated uncertainty. However, it is possible to take into account this contribution in the method’s calculations when necessary. For low volatility liquid sorbents with high viscosities, such as ionic liquids, samples were initially kept under vacuum for up to 15 min to ensure thorough initial degassing that can be limited by mass transport.

After purging and evacuation, the vials containing sorbent are pressurized with the selected gas or gas mixtures to an “initial” pressure (*p*_*ini*_) measured by the pressure transmitter (see Figures 1 and 2). After the pressure in the vials stabilizes (15–50 s depending on the needle), they are immediately removed from the gas system.

For porous solid and chemisorbent materials (such as aqueous MEA), gas uptake kinetics are typically more rapid than those for liquid physisorbents, making it more difficult to estimate an “initial pressure” of the measurement vials. The combination of the right needle internal diameter and freezing of the liquid minimizes these difficulties. Furthermore, we compared the pressure obtained in calibration vials with the estimated pressure obtained in these samples in the exact same conditions, and the pressure variability falls within the method’s usual range.

As the samples are equilibrated *ex situ* and not monitored during this period, it was crucial to determine the minimum equilibration time to ensure precise, consistent, and reproducible results. This was determined by producing samples in triplicate with periodic determination of the headspace peak area until a constant value was reached, *i.e.*, thermodynamic equilibrium was reached. For the materials described here, equilibration times varied between a few minutes and 5 days.

After measurement, sorbents with negligible volatility (solids and ionic liquids) could be regenerated by heating overnight at 40 °C under reduced pressure (100 mbar) in vented vials. Full regeneration was demonstrated by repeat gas loading with equivalent gas uptakes in subsequent measurements within the expected uncertainty.

We initially screened CO_2_ and CH_4_ uptake in a range of sorbents, along with C_2_H_4_/C_2_H_6_ in a chemisorbent ionic liquid to validate the method. We chose six representatives of organic solvents: dimethyl sulfoxide, polyethylene glycol 200, glycerol, ethylene glycol, octanol, and sulfolane; two ionic liquids: [C_4_C_1_Im][NTf_2_] and [C_6_C_1_Im][NTf_2_]; a porous liquid (PL) formed by combination of Genosorb 1753 with 30 wt % zeolite RHO: two chemisorbent liquids: [C_4_C_1_Im][NTf_2_] containing 0.25 mol L^–1^ Ag[NTf_2_], IL_Ag_ (offering complexation to C_2_H_4_), 30 wt % aqueous methanolamine, MEA (a CO_2_ chemisorbant), and solid 3 and 4 Å molecular sieves (MS3 and MS4, respectively) and zeolite RHO (PS) were investigated.

**Figure 4:**
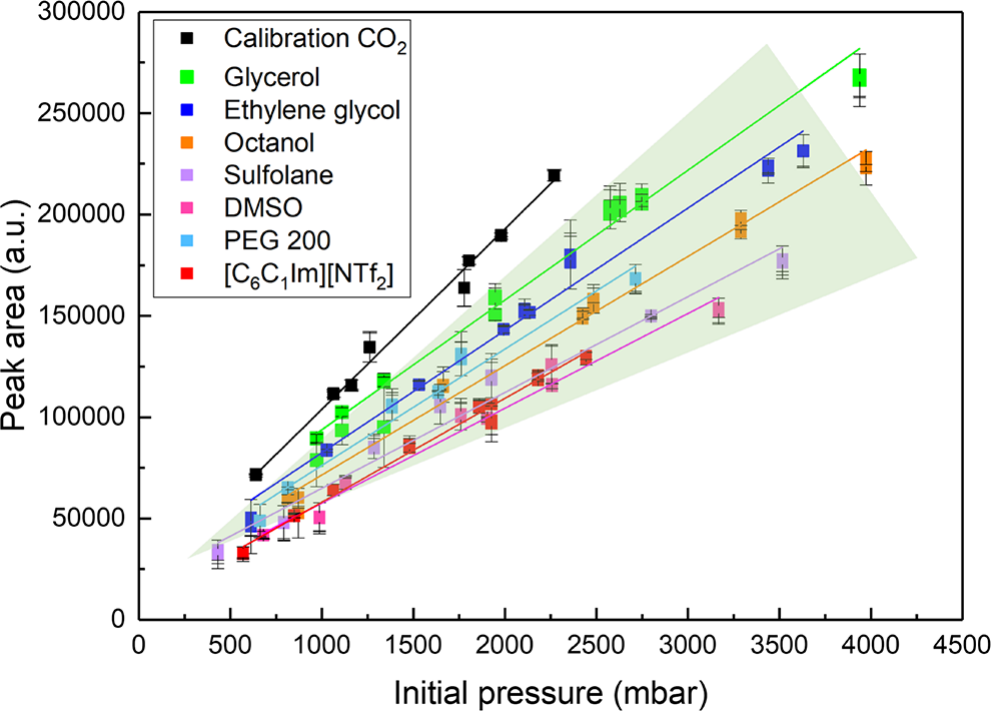
GC peak area obtained from CO_2_ equilibrated sorbent samples at pressures (*p*_ini_) between 500 and 4000 mbar at 308 K. Measurements were obtained with 5 mL of vial volume occupied by glass beads (black, calibration curve) or by liquid samples (all others) after 5 days of equilibration, showing clearly the decrease in peak area due to gas uptake by sorbent. Error bars represent the standard deviation of the fit for the data.

In this section, we present and discuss some of the CO_2_ uptake measurements for the organic solvents and ionic liquid (see Figure 4). All data and remaining plots for other sorbents can be found in the Supporting Information.

Results from screening CO_2_ with polyethylene glycol 200, glycerol, ethylene glycol, octanol, sulfolane, and the ionic liquid [C_6_C_1_Im][NTf_2_] using 5 mL of sorbent are shown in Figure 4 as a comparison of the peak area of CO_2_ equilibrated samples at pressures up to 2500 mbar and 308 K with the initial gas pressure, *p*_*ini*_, along with the corresponding calibration curve for a vial containing an equivalent volume of glass beads. Compared to the calibration line, in each case, the decrease in peak area observed for each sample as a function of *p*_*ini*_ can only be attributed to the absorption of gas into the sorbent material. Sorbents with higher CO_2_ absorption capacity such as DMSO and sulfolane can be clearly differentiated from those with lower capacities such as glycerol and ethylene glycol^19−23^ by the size of the peak area reduction.

Comparable results from screening CH_4_ uptake in polyethylene glycol 200, glycerol, ethylene glycol, octanol, DMSO, and sulfolane were obtained, although the reductions in peaks below those of the calibration line were significantly reduced as a consequence of lower CH_4_ solubility in these liquids (Figure S4). Changing the sorbent:head space volume ratio by increasing the amount of sorbent to 10 mL, increased gas absorption and resultant decrease in the peak areas for each *p*_*ini*_ were observed with ethylene glycol, octanol and [C_6_C_1_Im][NTf_2_] (Figure S5). The small CH_4_ capacities yield large errors in the data, especially at smaller pressures. We can distinguish octanol having a greater gas uptake capacity compared with either [C_6_C_1_Im][NTf_2_] or ethylene glycol, in agreement with literature,^24−26^ but it is impossible to clearly distinguish all the materials tested using this method due to their similar and low uptake capacities. However, determining that these materials offer very modest CH_4_ uptake capacities is the main goal of this screening method instead of a very precise uptake measurement.

Error bars were determined from the standard deviation of the fit for the data or their average absolute deviations. All these, the linear correlation fits, and correlation coefficients (*R*^2^) for each system along with measurement repeatability assessments are reported in the Supporting Information.

### Benchmarking and Comparison with the Literature

**Figure 5:**
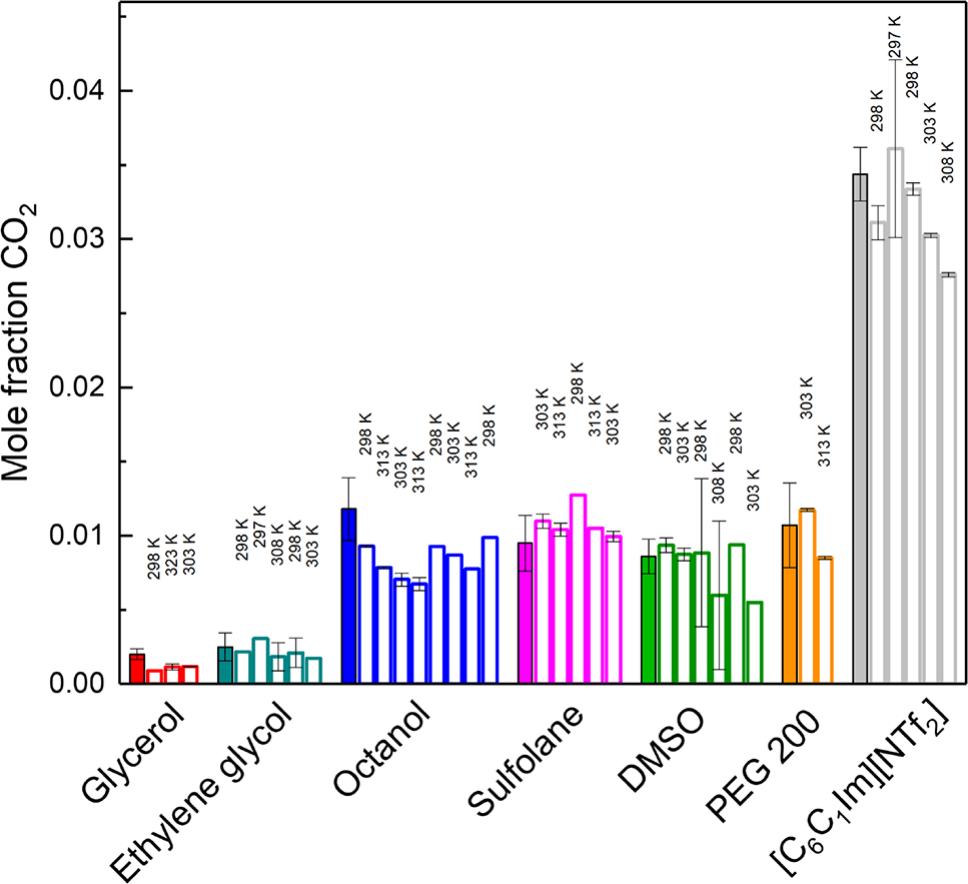
CO_2_ absorption results, in mole fraction of gas, obtained from HS-GC method at 308 K (solid bars) and those published in the literature at 303 K (open bars), at 1000 mbar, for selected organic solvents.^19−23,27,28^ Calibration and measurements were obtained using 5 mL of glass beads or liquid sorbent, correspondingly. Error bars represent the average absolute deviation of the fit of the corresponding data. *, Glycerol at 323 K.

The results shown in Figure 4 demonstrate a reduction in the amount of gas (CO_2_ in this case) present in the headspace above the sorbents, which is consistent with gas uptake into the liquid sorbents. After converting the sorbent capacity from eq 5 into mole fraction of gas, a comparison of CO_2_ absorption results at 1000 mbar partial pressure at equilibrium at 308 K collected here with the extant literature at comparable temperature and pressure^19−23,27,28^ can be made. This comparison is shown in Figure 5 showing data from the studies here at 308 K and literature data between 298 and 213 K as available.

CO_2_ uptake data from the HS-GC screening method compares well with results from the literature taken using a range of methods, with the smallest mole fraction CO_2_ solubility in glycerol followed by ethylene glycol. Similar mole fractions of CO_2_ are adsorbed by dimethyl sulfoxide, sulfolane, octanol, and polyethylene glycol 200, with the ionic liquid [C_6_C_1_Im][NTf_2_] showing the largest molar uptake of CO_2_ at 1000 mbar pressure at equilibrium. The values are in good agreement with the literature within the established uncertainties with the exception of glycerol. However, it should be noted that absorption capacities for CO_2_ in glycerol are small and that the literature contains values that are variable, potentially as an impact of the presence of water as a contaminant in these hygroscopic liquids. Determination of the water content is not always disclosed in the literature samples, as was the case for glycerol.

Uptake measurements performed with C_2_H_4_ in the ionic liquid [C_4_C_1_Im][NTf_2_] with this method show that our results also fall well within four of the five reference data sets obtained in the relevant literature, for the established uncertainties (Figure S6). The results obtained by Zhang et al.^29^ represent the lowest solubility values of the set and are the only one that diverges from our data. However, the authors do not report the uncertainty associated with their measurements or the water content of the ionic liquid. It is also worth mentioning that these results were obtained from GC across an IL-packed column, while all the other referenced works are based in less intrusive volumetric or gravimetric techniques.

**Figure 6:**
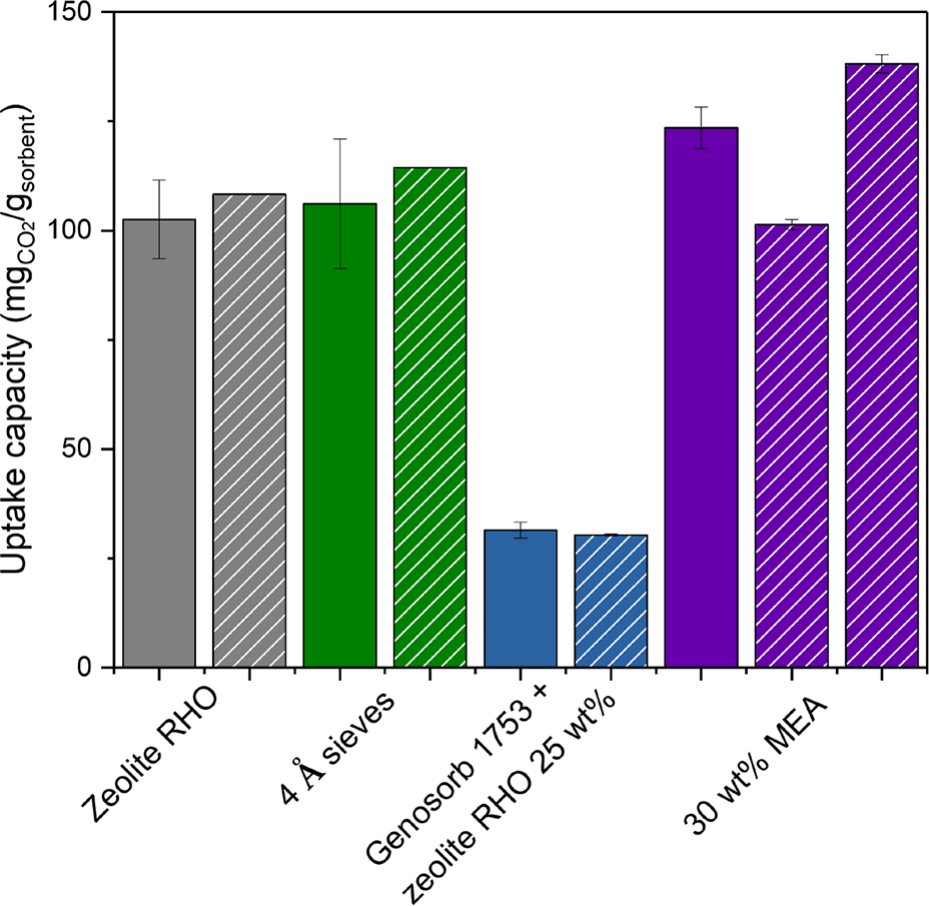
CO_2_ sorption results, in mg of gas per g of sorbent, obtained from HS-GC method at 308 K (solid bars) and those published in the literature at 303 K (striped bars), at 1000 mbar, for selected solid sorbents,^30^ a porous liquid,^31^ and a 30% weight monoethanolamine aqueous solution (MEA).^32,33^ Measurements were obtained with approximately 0.5 g of solid sorbents and 1 mL of liquid sorbents. Empty vials were used for the calibration curves. Error bars represent the average absolute deviation of the fit of the corresponding data.

CO_2_ uptake was then examined with high capacity solid sorbents (zeolite RHO and 4 Å molecular sieves), the porous liquid (zeolite RHO 25 wt % in Genosorb 1753) and chemisorbant 30 wt % aqueous monoethanolamine (MEA). The results and literature data taken under comparable conditions^34^ are shown in Figure 6 shown as uptake capacities. A high uptake capacity for CO_2_ was recorded for 30 wt % aqueous MEA (125 mg_CO2_/g_sorbent_) which is intermediate between the various values reported in the literature.^32,33^ High gas uptakes with zeolite RHO and 4 Å molecular sieves obtained were also consistent with reported values,^30^ as were the results with the porous liquid (Genosorb 1753+zeolite RHO)^31^ with capacity of approximately a quarter of that from the corresponding bulk zeolite which is consistent with gas absorption into the zeolite structure rather than in the polyethylene glycol dialkyl ether supporting liquid.

### Determination of Real Gas Selectivity

An advantage of using HS-GC as a quantification method is that it allows for the separation and identification of multiple components in the headspace sample, something that is not frequently found in the relevant literature. Mixtures of gases can be used for the direct determination of the ability of a sorbent to capture specific gases from mixtures or in the presence of contaminants representing more realistic gas capture conditions. This is particularly important for systems where the competitive nature of gas sorption may lead to a selectivity different from that of the calculated ideal. Selectivity in gas mixtures was examined for two exemplar systems of interest, CO_2_ and CH_4_ and C_2_H_4_ and C_2_H_6_, using sulfolane and a silver salt containing ionic liquid, respectively, to demonstrate applicability for a range of gas separation challenges.

**Figure 7:**
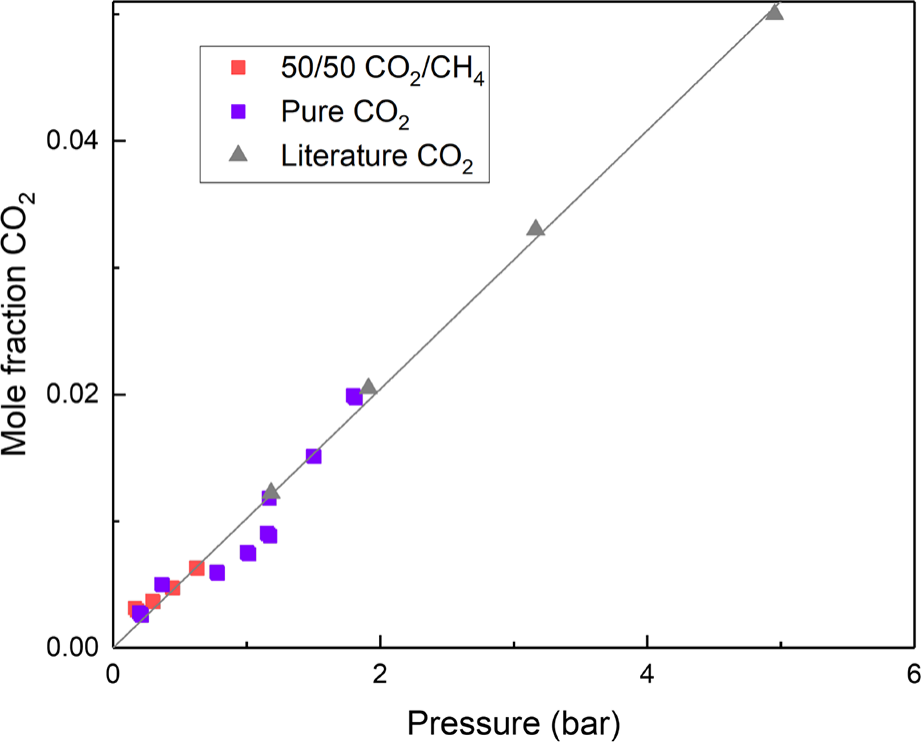
CO_2_ mole fraction solubility in sulfolane at pressures between 1 and 5 bar. Results from this work (square symbols) obtained with pure CO_2_ (purple) and 1:1 molar CO_2_/CH_4_ gas mixture (red) are represented along with the literature (gray triangles - pure CO_2_).^20^ Results obtained using the screening method were carried out at partial pressures of CO_2_ between 200 and 2000 mbar at 308 K using 5 mL of HS-GC occupied volume. Literature results at 303 K.

In Figure 7, we can see the data obtained for the CO_2_ uptake capacity with sulfolane, with both pure CO_2_ and its 1:1 molar mixture with CH_4_. These results are compared with the literature data for pure CO_2_ uptake.^20^

**Figure 8:**
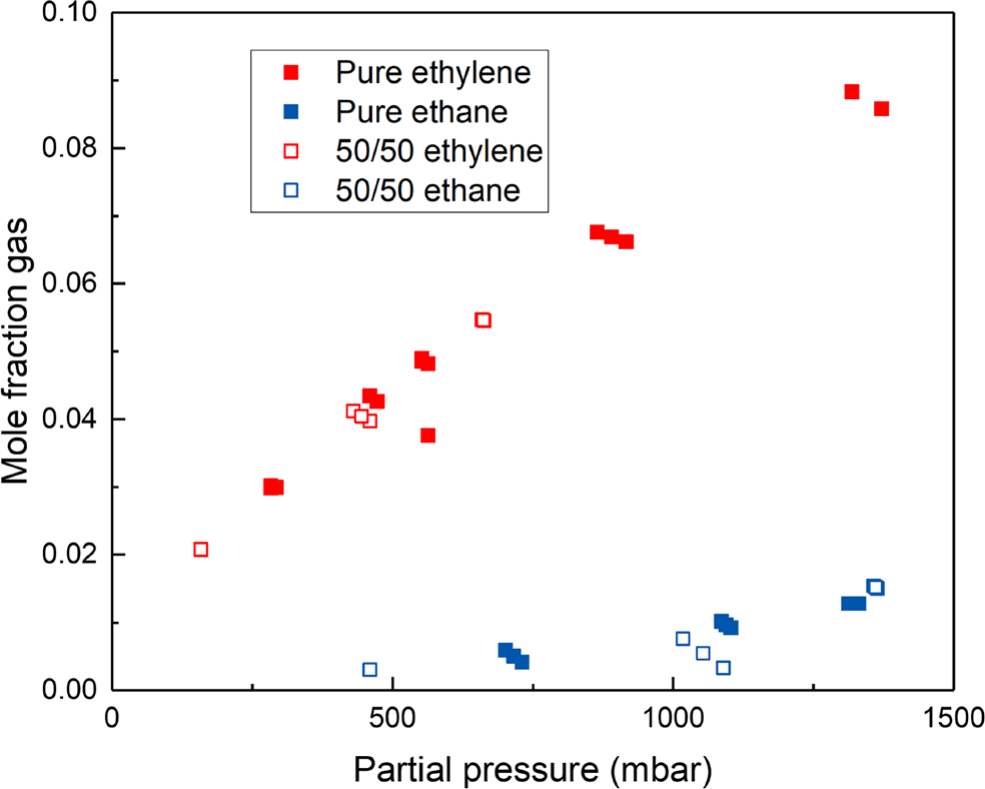
Ethylene, C_2_H_4_ (red squares), and ethane C_2_H_6_ (blue squares) mole fraction solubility in the ionic liquid [C_4_C_1_Im][NTf_2_] containing 0.25 mol L^–1^ concentration of Ag[NTf_2_] at pressures/partial pressures between 200 mbar and 1500 mbar obtained using the HS-GC method, at 308 K. Measurements were carried out using both pure gases (solid symbols) and 50/50 mixed gases (empty symbols). All measurements were performed using 3 mL of HS-GC vial occupied volume.

We observe that as expected for a physisorbant such as sulfolane, the impact of using a gas mixture instead of a pure gas steam is minimal, particularly at such low pressures. Similar experiments were carried to determine the C_2_H_4_ and C_2_H_6_ pure and mixed gas solubility in [C_4_C_1_Im][NTf_2_] ionic liquid containing 0.25 mol L^–1^ concentration of Ag[NTf_2_]. The results are presented in Figure 8 for a range of pressures. As expected, since the major source of ethylene uptake capacity is through complexion with silver, there is no competitive mechanism for the absorption, leading to almost identical uptake capacities for both gases in both cases.

**Figure 9:**
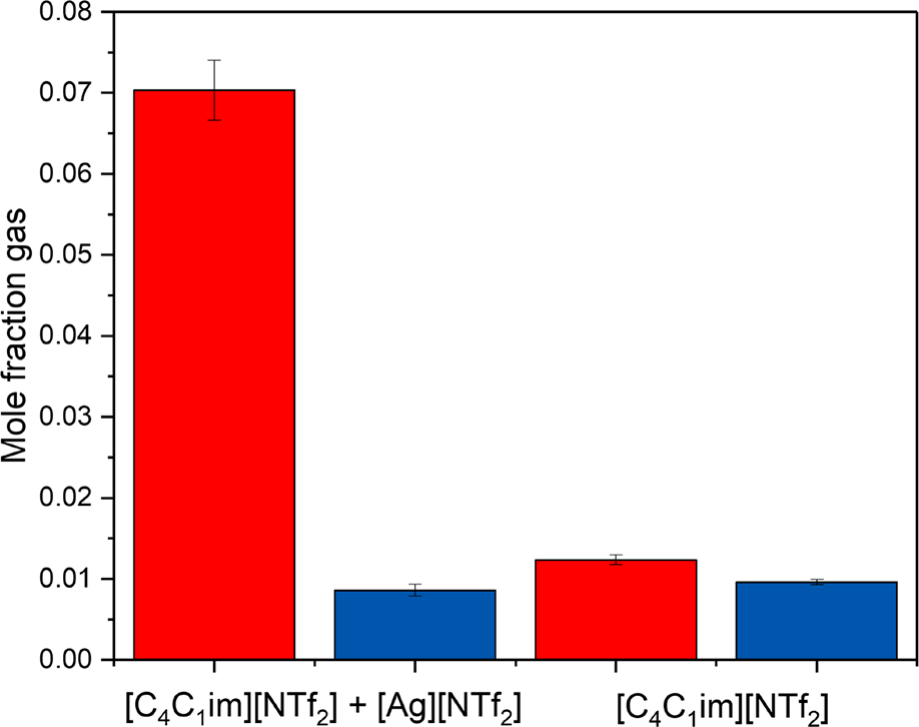
C_2_H_6_ (blue bars) and C_2_H_4_ (red bars) pure gas absorption results, in gas mole fraction, obtained from the HS-GC screening method at 308 K (solid bars) at 1000 mbar, for pure [C_4_C_1_Im][NTf_2_] (right), and for [C_4_C_1_Im][NTf_2_] + Ag[NTf_2_] (left) for a silver concentration of 0.25 mol L^–1^. Measurements/calibration were performed using 3 mL of sorbent/glass beads. Error bars represent the average absolute deviation of the fit of the corresponding data.

We then compared these results with the absorption of the pure gases in the ionic liquid before the silver addition (Figure 9) at 1000 mbar and 308 K. The results show similar and relatively low absorption of both gases in the pure ionic liquid, and a 5-fold increase in ethylene uptake, and selectivity from approximately 1.3 to 8.2 upon addition of silver salt to the sorbent. These results are in good agreement with literature where ideal selectivity values increased by almost 8-fold, from approximately 1.37 to 10.7 for the same chemisorbent ionic liquid at the same silver salt concentration and 560 mbar ethylene pressure at 303 K.^35^

The real selectivity obtained, 9.2, is close to the ideal selectivity described in the literature and those obtained from our own results, as expected for these systems in mild conditions.

### Sources of Error and Method Limitations

Several possible sources of error were identified in association with the method. The most relevant is likely the temperature control. As the samples vials are being pressurized, they are conditioned at room temperature, which is subject to variation. After equilibration at controlled temperature, the vials are transferred to the HS-GC at room temperature and equilibrated again in the HS-GC oven (with no stirring) at controlled temperature for 2 h. These temperature variations justify, at least in part, both the dispersion and deviation of our measures relative to those in the known literature. As expected, the dispersion of the data in the calibration curve and isotherms leads to error propagation within our calculations. Other smaller sources of error can come from variations in the HS-GC vial volume and variations in the glass beads and stir bar volumes.

The level of uncertainty that we report is sufficient for a clear comparison and ranking even between materials within the same range of gas solubilities. The goal of this screening method is to streamline the selection and testing of materials for gas capture and separation, saving resources and research time. It was not designed to produce high accuracy results, and although implementing further steps to increase its precision and accuracy could be taken, it would certainly negatively affect its throughput.

We estimate that the minimum pressure variation in the HS-GC vials that can be used with this method should be above 65–70 mbar, corresponding to the uptake of approximately a minimum of 0.04 mmol or 1.8 mg of CO_2_. For the ionic liquid [C_6_C_1_Im][NTf_2_], as an example, this corresponds to a lower mole fraction of 0.0027. This estimation is based on the minimum pressure difference needed between the calibration curve and measurement to obtain a reliable and reproducible result, above the corresponding average absolute deviations.

The results obtained with the least amount of uncertainty were, as expected, for materials with high gas uptake, since a larger difference between the calibration curve and the measurement leads to a more reliable result. For materials with low gas uptake, we recommend increasing the amount of sorbent, increasing the initial gas pressure, and/or miniaturizing the procedure with 10 mL HS-GC vials.

Since this method is based on HS-GC it shares its limitations. Certain gases or mixtures might be too complex to analyze, notoriously mixtures with SO_2_, other very reactive gases, or impurities that may react with GC internal parts or that easily degrade at the GC internal temperatures. Some gas mixtures may call for the use of 2 columns or a variety of GC detectors. However, the usual GC detectors present wide gas detection range and detection limits well below the concentrations used in this method.

Although the HS-GC vials used here are rated for 5 bar, for safety reasons, we do not recommend using pressures well above atmospheric if not necessary and never with nonspecked vials. The transportation of glass vials under the higher pressure range, particularly containing glass beads, should be done with particular care.

### Advantages and Benefits

Traditional gravimetric or volumetric methods or even a Brunauer–Emmett–Teller (BET) analysis requires between 1 day and 1 week per measurement per sample. As in the methods above, the slow step in this method remains the equilibration time of the gas with the sorbent, representing a few minutes for some microporous solids up to 5 days with viscous ionic liquids. However, by offering *ex situ* equilibration, we bypass this challenge, allowing for the production of one set of triplicate samples every 5–15 min. This means that a long equilibration time for one sample does not impact the production or analysis of others.

Considering the 3–15 min of vacuum applied before the sample pressurization and 1 min of pressurization, we can produce between 30 and 96 triplicate samples per day, the lower limitation being the slow vacuum stage that was expedited with the wider trident needles. If only single measurements are required, then between 90 and 264 different sorbents can be tested in the same amount of time. This estimate takes into account the 2 h equilibration time in the GC oven and a 5 min GC analysis run. From this we estimate a 90–3000 times reduction in the time required for equivalent analysis.

An additional benefit is that the method is adaptable to a range of uptake capacities and a wide variety of materials, solids and liquids, volatile and nonvolatile, and chemisorbent and physisorbent.

We can simultaneously and directly screen the sorbents for their gas separation ability by using gas mixtures. This is highly relevant for materials that, for example, contain metal sites that may interact with different gases in the mixture, such as MOFs and many porous liquids or other chemisorbent materials.

Finally, one additional benefit of this method is that it does not require specialized equipment, since a HS-GC is frequently present in research and industrial laboratories, opening the screening of materials for gas uptake and separation for a wide range of research(ers).

## Conclusions

We have developed a high-throughput gas uptake screening approach suitable for liquid and solid sorbents that is based on headspace gas chromatography. We have proven that this universal method can be applied with pure and mixed gas feeds for fast, universal experimental screening of gas uptake and determination of real gas separation selectivities across a range of pressures and temperatures. This method allows for screening of both solid and liquid materials, volatile or nonvolatile, physisorbent or chemisorbent, combined with pure or mixed gas streams. As an example, we can adequately detect CO_2_ sorption for a minimum gas uptake of 0.0027 in mole fraction in [C_6_C_1_Im][NTf_2_]. This universal method is also adaptable to a wide range of gas uptake capacities, and although the slowest step in the process remains the equilibration, the ability to “number up” samples for off-line equilibration overcomes this restriction, allowing for the screening of up to 264 different sorbents per day. Other gases of relevance can be studied using this technique, such as NH_3_, H_2_, and SO_2_, although some may require more specialized gas chromatography analytics than a standard FID detector. For example, we have extended this experimental methodology to H_2_ quantification, which should soon follow this publication.

## Supporting Information Available

Experimental data on gas sorption measurements, HS-GC calibration curves and characterization data (PDF)
