# Supplementary material for: A High-Throughput Experimental Approach to Screening Gas Sorption by Liquids and Solids
Source: ACS Sustain Chem Eng. 2023 Dec 7;11(50):17787–96. doi: 10.1021/acssuschemeng.3c05901 (PMC10731633; doi:10.1021/acssuschemeng.3c05901)
Supplement: Supplementary file 1 — sc3c05901_si_001.pdf [file sc3c05901_si_001.pdf]

A High-Throughput Experimental Approach to Screening Gas Sorption by Liquids and Solids

J. Mark Young,† Sam H. McCalmont,† Sophie Fourmentin,‡ Panagiotis Manesiotis,¶ John D. Holbrey,† and Leila Moura*,†

†*QUILL Research Centre, Queen's University Belfast, School of Chemistry and Chemical Engineering, David Keir Building, 39-123 Stranmillis Road, Belfast, BT9 5AG, United Kingdom*

‡*Unité de Chimie Environnementale et Interactions sur le Vivant (UCEIV), EA 4492 SFR Condorcet FR CNRS 3417, Université du Littoral-Côte d'Opale, 59140, Dunkerque, France*

¶*Queen's University Belfast, School of Chemistry and Chemical Engineering, David Keir Building, 39-123 Stranmillis Road, Belfast, BT9 5AG, United Kingdom*

E-mail: l.moura@qub.ac.uk

Supplementary Information for Publication

Pages-42

Figures-6

Tables-42

Equipment information

Density and Viscosity

The Anton Paar SVM 3001 is used for both density and viscosity. The accuracy and precision of the densimeter were tested before the measurements with certified ultra-pure water and. The precision of the density results are estimated to be in the order of magnitude of $0.01\,\mathrm{kg}\cdot\mathrm{m}^{-3}$ and the accuracy is estimated to be better than $0.01\,\%$. The temperature is controlled within $\pm 0.005\,\mathrm{K}$. The precision of the viscosity results are estimated to be in the order of magnitude of $0.01\,\mathrm{kg}\cdot\mathrm{m}^{-3}$ and the accuracy is estimated to be better than $0.01\,\%$. The temperature is controlled within $\pm 0.005\,\mathrm{K}$.

Water content

The amount of water contained in each ionic liquid after the drying process was determined by a coulometric Karl Fisher titrator (Metrohm 899 Coulometer using Hydranal Coulmat AG-H as reagent).

Mass Spectroscopy

The high-resolution mass spectra (MS-Q-TOF) were recorded in positive and negative mode on a hybrid quadrupole time of flight (TOF) mass spectrometer (Waters Xev0 G2-XS Qtof) with an Electrospray Ionisation (ESi) source. The gas flow of spray gas is 50 l/hour, capillary voltage : +/- 3.0 kV. Cone voltage : 50 V and source temperature : 150 °C Mass range: 20-1600 m/z for positive and negative modes

Synthesis and characterisation of sorbent materials

Synthesis of $[C_4C_1Im]Cl$

1-Chlorobutane (128.79 g, 1391 mmol) was added to freshly distilled 1-methylimidazole (70.50 g, 859 mmol) . The mixture was stirred for 48 hours at 65 °C in acetonitrile, then

concentrated by rotary evaporation. The product was dried overnight under vacuum giving a white powder (89 g, 81.23 %) ^{1}H-NMR (400 MHz, $DMSO_4$) δ (ppm): 9.10 (1H, s, N−C**H**−N), 7.74 (2H, m, N−C**H**−C**H**−N), 4.16 (2H, t, N−C$\mathbf{H_2}$−CH_3), 3.85 (3H, s,C$\mathbf{H_3}$−N), 1.77 (2H, m,CH_2−C$\mathbf{H_2}$−CH_2−CH_3), 1.28 (2H, m, CH_2−C$\mathbf{H_2}$−CH_3), 0.90 (3H, t, CH_2−C$\mathbf{H_3}$). ^{13}C-NMR (400 MHz, $DMSO_4$) δ (ppm): 136.2 (s, N−**C**H−N), 124.6 (s, N−**C**H−CH−N), 121.2 (s,N−CH−**C**H−N), 49.8 (s, N−$\mathbf{C_2}$), 36.6 (s,N−**C**H_3), 32.9 (s, N−CH_2−$\mathbf{C_2}$), 19.2 (s,−CH_2−**C**H_2−CH_3), 13.2 (s, −CH_2−**C**H_3).

Synthesis of [C_4C_1Im][NTf_2]

1-Butyl-3-methylimidazolium bis(trifluoromethylsulfonyl)imide ([C_4C_1Im][NTf_2]) was synthesised based on previous reports.[1] Li[NTf_2] (38 g, 172 mmol) was dissolved into 50 ml of deionised water, and [C_4C_1Im]Cl (29 g, 166 mmol) dissolved into 50 ml of deionised water. Both solutions were mixed, and stirred for 24 hours at room temperature. Dichloromethane (60 ml) was added to the mixture, and the mixture was then transferred to a separating funnel. The lower organic phased was collected and washed with water. This was repeated until no traces of halide anion could be seen in the aqueous phase (tested with silver nitrate solution). The solvent was removed by vacuum giving a colourless liquid. ^{1}H-NMR (400 MHz, $DMSO_4$) δ (ppm): 9.47 (1H, s, N−C**H**−N), 7.78 (2H, m, N−C**H**−C**H**−N), 4.19 (2H, t, N−C$\mathbf{H_2}$−CH_3), 3.87 (3H, s,C$\mathbf{H_3}$−N), 1.76 (2H, m,CH_2−C$\mathbf{H_2}$−CH_2−CH_3), 1.23 (2H, m, CH_2−C$\mathbf{H_2}$−CH_3), 0.89 (3H, t, CH_2−C$\mathbf{H_3}$). ^{13}C-NMR (400 MHz, $DMSO_4$) δ (ppm): 137.2 (s, N−**C**H−N), 123.6 (s, N−**C**H−CH−N), 122.7 (s,N−CH−**C**H−N), 118.3 (s, -**C**F3) 48.5 (s, N−$\mathbf{C_2}$), 35.7 (s,N−**C**H_3), 31.3 (s, N−CH_2−**C**H_2), 19.1 (s,−CH_2−**C**H_2−CH_3), 13.2 (s, −CH_2−**C**H_3).

Synthesis of [C_6C_1Im][NTf_2]

1-hexyl-3-methylimidazolium bis(trifluoromethylsulfonyl)imide ([C_6C_1Im]Br) was kindly donated by the QUILL Research Centre, having been synthesized as follows: Li[NTf_2] (112 g,

393 mmol) was dissolved into 50 ml of deionised water and stirred with $[C_6C_1Im]Br$ (78.3 g, 357 mmol) for 24 hours at room temperature. Dichloromethane (60 ml) was added to the mixture. The lower organic phased was collected and washed with water. This was repeated until no traces of halide anion could be seen in the aqueous phase (tested with silver nitrate solution). The solvent was removed by vacuum giving a pale yellow liquid. ^{1}H-NMR (400 MHz, CD_3Cl) δ (ppm): 8.67 (1H, s, N−C**H**−N), 7.31 (2H, m, N−C**H**−C**H**−N), 4.13 (2H, t, N−C$\mathbf{H_2}$−CH_3), 3.90 (3H, s,C$\mathbf{H_3}$−N), 1.83(2H, m,CH_2−C$\mathbf{H_2}$−C_4H_9), 1.28 (2H, m, CH_2−$C_3$$\mathbf{H_6}$−$CH_3$), 0.85 (3H, t, CH_2−C$\mathbf{H_3}$).

^{13}C-NMR (400 MHz, $DMSO_4$) δ (ppm): 135.9 (s, N−**C**H−N), 123.7 (s, N−**C**H−CH−N), 122.3(s,N−CH−**C**H−N), 119.8 (s,**C**F_3), 50.1 (s, N−**C**H_2), 36.2 (s,N−**C**H_3), 30.9 (s, −CH_2−$\mathbf{C_2}$-CH2), 30.0 (s, −CH_2−$\mathbf{C_2}$-CH2), 25.6 (s, −CH_2−$\mathbf{C_2}$-CH2), 22.3 (s,−CH_2−**C**H_2−CH_3), 13.8 (s, −CH_2−**C**H_3).

Synthesis of Ag[$\mathbf{NTf_2}$]

Ag[NTf_2] was synthesised *via* the procedure reported by Agel *et al.*[2] Silver nitrate (4.50 g, 26.512 mmol) was added to 15 ml of distilled water. A sodium hydroxide solution (20 w%) was added drop-wise until the mixture recorded a pH of 11.3. A dark brown precipitate was formed, filtered, and washed with distilled water. The precipitate was then added to a 15 ml solution of bis(trifluoromethylsulfonyl)imide H[NTf_2] (7.3772 g, 26.239 mmol)(0.99 molar equivalent of silver). The mixture is allowed to stir for 2 hours at 60 °C, then cooled. The excess AgOH is filtered off at room temperature. The produce was then dried at 50 °C under high vacuum. Ag[NTf_2] was obtained as a white solid. (υ_{max}/cm^{-1}): 733, 792, 1016, 1103, 1193, 1307, 1329. *m/z* (ESI-MS negative mode): 281.91 NTf_2^-, 666.72 Ag[NTf_2], 668.72 Ag[NTf_2].

Table S 1: Water content of the liquid sorbents, in ppm

Sorbent	Amount of water /ppm
$[C_4C_1Im][NTf_2]$	<200
$[C_6C_1Im][NTf_2]$	650
DMSO	1585
Sulfolane	3894
Glycerol	1733
Ethylene glycol	2172
Octanol	1667

Water content

IR Spectroscopy

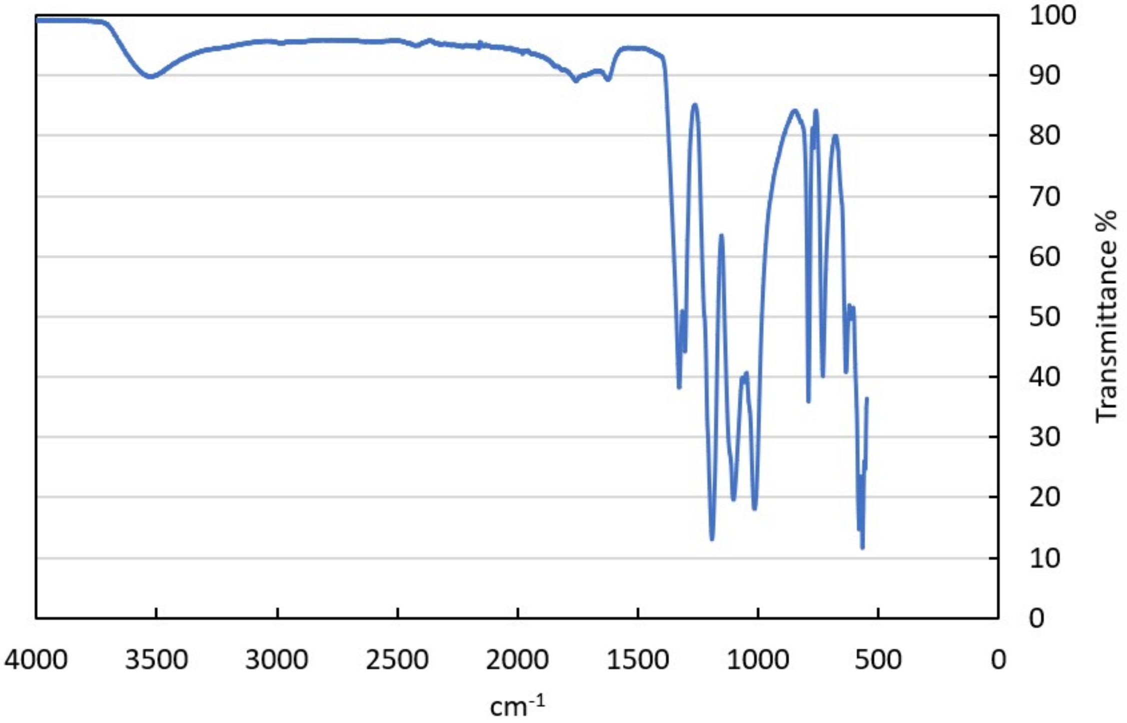

Figure S 1: IR-spectrum of $Ag[NTf_2]$

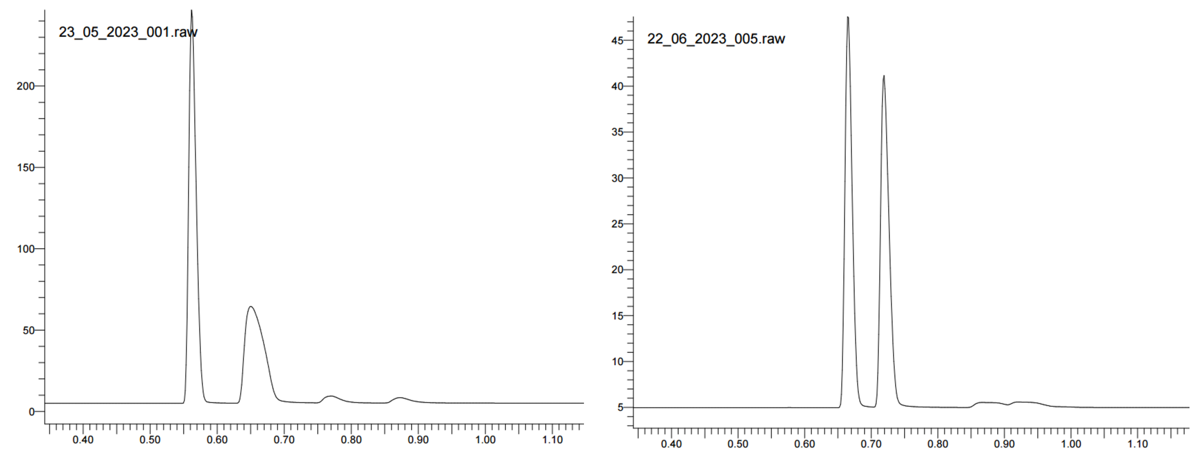

Figure S 2: Headspace gas chromatograms for CO_2/CH_4 (left) and C_2H_6/C_2H_4 (right) gas mixtures of with elution times for CH_4, CO_2, C_2H_6 and C_2H_4 at 0.57, 0.68, 0.66 and 0.72 minutes, respectively.

Calibration and solubility data

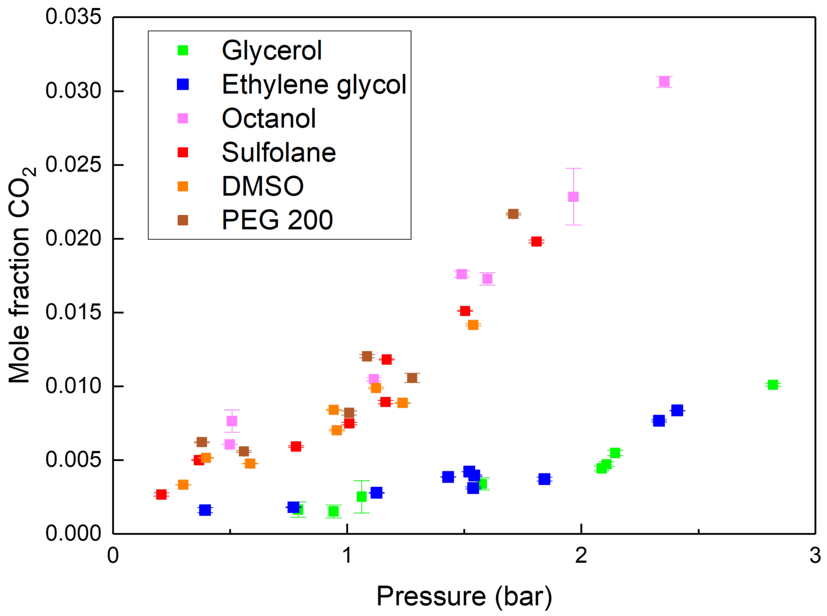

Figure S 3: Mole fraction solubilities of CO_2 in equilibrated sorbent samples at pressures between 300 mbar and 3000 mbar at 308 K. Measurements were obtained with 5 ml of vial volume occupied by liquid samples after 5 days of equilibration and calculations were carried out based on calibration curves using 5 ml glass beads in vials. This data clearly shows that clear differences can be seen between the gas capacity of the materials.

Table S 2: Calibration data for CO_2, CH_4 and CO_2/CH_4 mixed gas using 5 ml of glass beads and the method using 23s G needles with 15 minute evacuation times where pressure is measured in mbar and peak area is expressed in a.u.

Gas						
CO_2		CH_4		CO_2/CH_4		
Gas pressure (mbar)	Peak area (a.u.)	Gas pressure (mbar)	Peak area (a.u.)	Total pressure	CH_4 peak area (a.u.)	CO_2 peak area (a.u.)
2270	219442	2591	323882	1989	136714	95150
2270	219081	2591	329210	1989	137689	96638
2270	219453	2591	330073	1989	138050	95157
1262	134408	692	99207	864	62315	46959
1262	134734	692	99666	864	62380	46843
640	71805	692	99703	864	62952	46701
640	71364	860	124606	1451	102575	75271
1980	189925	860	123788	1451	103211	74161
1980	189343	860	124303	1451	102226	75235
1804	177170	2424	310625	1579	111172	78903
1804	177256	2424	311948	1579	111306	79229
1065	111194	2424	311122	718	51554	39439
1065	111730	2681	341060	718	51462	39349
1162	115750	2681	340027	2532	171510	115747
1162	115577	2681	337768	2532	170314	115266
1162	116062	3133	372195			
3334	257952	3133	372561			
3334	258173	3133	371089			
4120	273271	3973	402082			
4120	273230	3973	411695			
4120	273508	3973	408750			
		3235	378909			
		3235	379581			

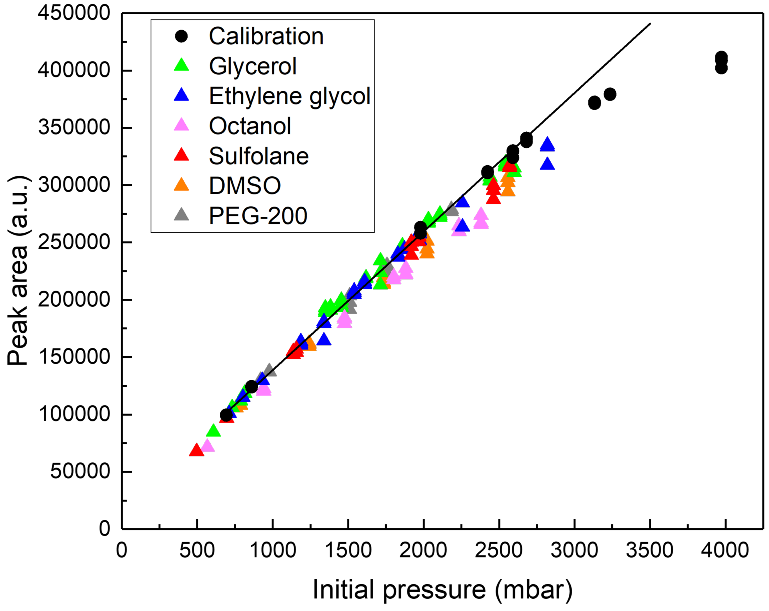

Figure S 4: GC peak area of CH_4 equilibrated samples at pressures (p_{ini}) between approximately 500 mbar to 4000 mbar at 308 K. Measurements were obtained with 5 ml of vial volume occupied by glass beads (black, calibration curve) or by liquid samples (all others) after 5 days of equilibration, in many cases showing decrease in peak area due to gas uptake by sorbent.

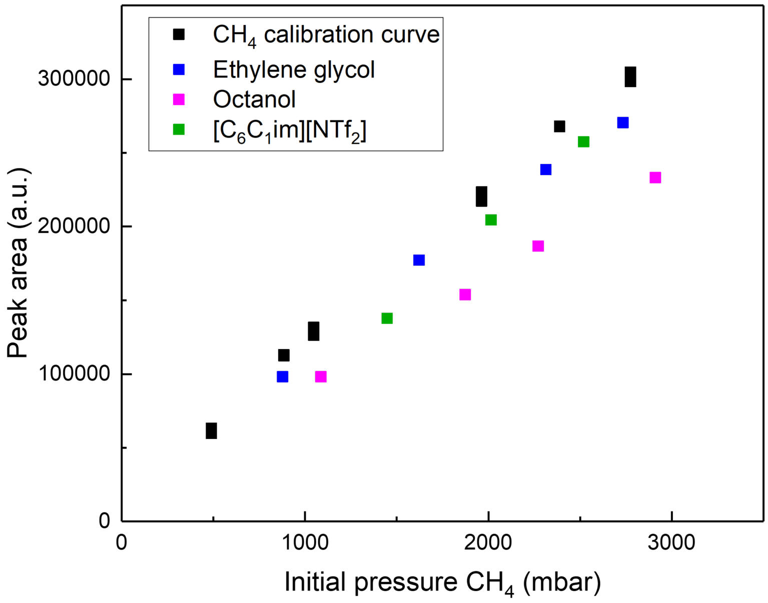

Figure S 5: CG peak area of CH_4 equilibrated samples at initial pressures between approximately 500 mbar to 3000 mbar at 308 K. Measurements were obtained with 10 ml of vial volume occupied by glass beads (black, calibration curve) or by liquid samples (all others) after 5 days of equilibration, showing clearly the decrease in peak area due to gas uptake by sorbent.

Table S 3: Calibration data for CO_2, CH_4 and CO_2/CH_4 mixed gas using 10 ml glass beads and the method using 20 G needles with 3 minute evacuation times where pressure is measured in mbar and peak area is expressed in a.u.

Gas				
CH_4		CO_2/CH_4		
Gas pressure (mbar)	Peak area (a.u.)	Total pressure	CH4 peak area (a.u.)	CO2 peak area (a.u.)
884	112991	3375	186340	118982
884	112584	3375	182990	117437
884	112386	3375	183328	117780
1963	223501	1975	117516	78722
1963	217214	1975	118187	79157
2775	303436	1975	122240	81543
2775	298288	1528	95595	65284
2775	304731	1528	97363	66309
2388	267634	1528	98527	66940
2388	267960	2427	139488	90556
490	63230	2427	141160	91566
490	59624	1261	77860	53479
490	62598	1261	77893	53553
1048	126347	848	53736	38054
1048	131549	848	54113	38287
1048	129759			
3842	365528			
3842	357266			
3842	366103			
3435	328375			
3435	343901			
3435	348379			

Table S 4: Calibration data for CO_2, CH_4 and CO_2/CH_4 mixed gas using empty vials and the method using 20 G needles with 3 minute evacuation times where pressure is measured in mbar and peak area is expressed in a.u. showing linearity for all gases until approximately 2500 mbar.

Gas			
CO_2		CH_4	
Gas pressure (mbar)	peak area (a.u.)	Gas pressure (mbar)	peak area (a.u.)
2829	225701	507	66430
2829	227064	507	63123
2829	226366	507	66350
1913	171624	927	123467
1913	169715	927	122773
1913	171173	927	126090
634	68485	1772	237495
634	68270	1772	241302
1787	162381	1772	241030
1787	163414	2130	282258
695	73664	2130	269976
695	73616	2130	266984
695	73778	2769	326363
1500	140954	2769	326680
1500	141108	2769	326535
1500	140650	3825	401796
2340	197988	3825	402616
2340	199131	3825	397986
2340	199502	4444	405585
2805	223767	4444	405162
2805	224652	4444	405618
2805	224894		

Table S 5: Calibration data for C_2H_4, C_2H_6 and C_2H_4/C_2H_6 mixed gas using 3 ml glass beads and the method using 20 G needles with 3 minute evacuation times where pressure is measured in mbar and peak area is expressed in a.u.

Gas						
C_2H_4		C_2H_6		C_2H_4/C_2H_6		
Gas Pressure (mbar)	peak area (a.u.)	Gas Pressure (mbar)	peak area (a.u.)	Total pressure	CH4 peak area (a.u.)	CO2 peak area (a.u.)
536	149019	500	123848	582	81594	81147
536	148340	500	144447	582	80482	80182
536	150383	500	142833	582	82391	81993
800	229109	796	225928	859	118773	117387
800	204236	796	224262	859	118779	117385
800	237027	796	223984	859	119155	117793
1028	311753	1112	310901	2014	267847	265098
1028	311661	1112	309225	2014	265486	262887
1028	269539	1469	404828	2014	267402	264961
1404	421078	1469	402238	1203	151564	160868
1404	412834	1469	401379	1203	153966	163422
1404	406127	1984	536212	1203	151204	160485
2964	754149	1984	538022	1549	192995	204921
2964	752916	1984	535840	1549	193716	205687
2964	735888	3110	815867	1549	194196	206198
3590	840160	3110	814993	2404	341712	338047
3590	839829	3110	803690	2404	328330	325413
3590	774460	3846	905214	2404	332304	329339
4104	863474	3846	897957	3262	431941	432865
4104	864584	3846	910124	3262	434994	435957
4104	863247	4615	902763	3262	438047	439049
4709	856383	4615	887923	4019	470468	474841
4709	853734	4615	883795	4019	463502	467165
4709	852441	2500	687369	4019	454853	458036
1854	500757	2500	676375	4600	438560	441047
1854	503224	2500	684583	4600	433506	435964
1854	503078			4600	393431	394266
2036	542897					
2036	549936					
2036	544759					
2526	656364					
2526	651316					
2526	652736					
2757	700701					
2757	705843					
2757	706245					

Table S 6: Parameters of a linear equation ($y = mx + c$) used to smooth the experimental results on peak area variation with the pressure of gas where x is pressure and y is peak area. Where M is the gradient of the fit, c is the y intercept of the fit, S.D. x_{gas} is the average standard deviation of the peak area from the fit in the Y estimate and R^2 is used as a measure of the variance of the data to the fit.

Gas/mixtures	m	c (a.u)	S.D	R^2
empty				
CO_2	71.64	28370	5175	0.992
CH_4	117.75	14925	12420	0.986
3 ml glass beads				
C_2H_4	243.32	40808	18375	0.992
C_2H_6	260.53	16547	11021	0.998
50/50 C_2H_4	269.37	-2407	8756	0.995
50/50 C_2H_6	266.96	1748	5032	0.998
5 ml glass beads				
CO_2	76.50	17579	3468	0.995
CH_4	119.78	18927	2487	1.000
50/50 CH_4 peak	131.87	5816	1340	0.999
50/50 CO_2 peak	84.48	11114	1926	0.995
10 ml glass beads				
CH_4	103.82	16987	4611	0.998
50/50 CH_4 peak	101.10	16317	3772	0.993
50/50 CO_2 peak	61.40	16377	2889	0.989

Table S 7: Data obtained for the solubility experiments using CO_2 in 4.8 ml glycerol where initial pressure (p_{ini}) is expressed in mbar, peak area is expressed in arbitrary units (a.u), p_{eq} is expressed in mbar, the repeatability (RPT) of these triplicate pressures is expressed in mbar, the gas capacity (C_{gas}) is expressed in mg/g, x_{gas} is the mole fraction of gas in the solvent and the mass of sorbent (m_{sorb}) is expressed in g.

CO_2						
Glycerol						
p_{ini} (mbar)	Peak area (a.u.)	p_{eq} (mbar)	RPT. p_{eq} (mbar)	C_{gas} (mg/g)	x_{gas}	m_{sorb} (g)
2628	204267	2110	19	2.26	0.004700	6.012
2628	201842	2083		2.37	0.004940	6.018
2628	205906	2128		2.19	0.004552	5.991
3937	268780	2829	12	4.83	0.010005	6.010
3937	268399	2825		4.86	0.010075	5.991
3937	266292	2802		4.94	0.010238	6.019
1110	93411	875	48	1.03	0.002143	6.012
1110	102494	976		0.59	0.001226	5.998
1110	102537	976		0.58	0.001219	6.010
2575	202684	2093	16	2.10	0.004385	6.007
2575	203611	2103		2.06	0.004291	6.007
2575	200208	2065		2.23	0.004648	5.990
1947	158558	1601	45	1.51	0.003139	6.030
1947	159600	1612		1.46	0.003053	5.994
1947	150537	1511		1.90	0.003964	6.004
972	89662	833	57	0.61	0.001272	5.997
972	89307	829		0.63	0.001308	5.996
972	78724	711		1.14	0.002377	6.010
1340	118303	1152	5	0.82	0.001715	6.000
1340	117429	1142		0.86	0.001799	6.018
2749	205302	2122	20	2.74	0.005700	6.000
2749	209633	2170		2.52	0.005251	6.015
2749	207031	2141		2.65	0.005515	6.012

Table S 8: Data obtained for the solubility experiments using CO_2 in 4.8 ml ethylene glycol where initial pressure (p_{ini}) is expressed in mbar, peak area is expressed in arbitrary units (a.u), p_{eq} is expressed in mbar, the repeatability (RPT) of these triplicate pressures is expressed in mbar,, the gas capacity (C_{gas}) is expressed in mg/g, x_{gas} is the mole fraction of gas in the solvent and the mass of sorbent (m_{sorb}) is expressed in g.

CO_2						
Ethylene glycol						
p_{ini} (mbar)	Peak area (a.u.)	p_{eq} (mbar)	RPT. p_{eq} (mbar)	C_{gas} (mg/g)	x_{gas}	m_{sorb} (g)
3630	231125	2410	2	5.99	0.0084	5.3432
3630	231231	2411		5.98	0.0084	5.3399
3630	231513	2414		5.96	0.0083	5.3446
1028	84123	771	4	1.26	0.0018	5.3603
1028	83434	763		1.30	0.0018	5.3450
1028	83366	763		1.30	0.0018	5.3528
2108	153430	1544	8	2.76	0.0039	5.3538
2108	151812	1525		2.85	0.0040	5.3485
2108	152182	1530		2.83	0.0040	5.3585
3439	224164	2332	12	5.43	0.0076	5.3456
3439	223763	2327		5.44	0.0076	5.3585
3439	221716	2305		5.56	0.0078	5.3478
2137	151448	1521	1	3.01	0.0042	5.3541
2137	151689	1524		3.00	0.0042	5.3495
2137	151739	1525		3.01	0.0042	5.3384
614	50290	394	23	1.07	0.0015	5.3690
614	46095	347		1.31	0.0018	5.3404
614	50582	397		1.07	0.0015	5.3351
1532	115952	1126	1	1.99	0.0028	5.3459
1532	116140	1128		1.97	0.0028	5.3751
1532	116092	1127		1.98	0.0028	5.3465
1993	143411	1432	1	2.75	0.0039	5.3535
1993	143200	1429		2.76	0.0039	5.3490
1993	143283	1430		2.76	0.0039	5.3368
2357	180289	1843	19	2.52	0.0035	5.3541
2357	176262	1798		2.74	0.0038	5.3495
2357	177163	1808		2.69	0.0038	5.3384
2002	152972	1538	11	2.26	0.0032	5.3690
2002	153609	1546		2.24	0.0031	5.3404
2002	155375	1565		2.15	0.0030	5.3351

Table S 9: Data obtained for the solubility experiments using CO_2 in 4.8 ml 1-octanol where initial pressure (p_{ini}) is expressed in mbar, peak area is expressed in arbitrary units (a.u), p_{eq} is expressed in mbar, the repeatability (RPT) of these triplicate pressures is expressed in mbar, the gas capacity (C_{gas}) is expressed in mg/g, x_{gas} is the mole fraction of gas in the solvent and the mass of sorbent (m_{sorb}) is expressed in g.

CO_2						
1-octanol						
p_{ini} (mbar)	Peak area (a.u.)	p_{eq} (mbar)	RPT. p_{eq} (mbar)	C_{gas} (mg/g)	x_{gas}	m_{sorb} (g)
1663	114859	1114	6	3.61	0.011	3.9930
1663	116047	1127		3.52	0.010	3.9922
1663	114916	1114		3.61	0.011	3.9824
3290	191314	1966	32	8.72	0.025	3.9816
3290	198292	2044		8.19	0.024	3.9871
3290	193572	1991		8.54	0.025	3.9840
814	59677	499	1	2.08	0.006	3.9840
814	59924	501		2.06	0.006	3.9846
814	59857	501		2.06	0.006	3.9804
3973	226199	2355	24	10.65	0.031	3.9827
3973	227956	2374		10.53	0.030	3.9801
3973	222813	2317		10.86	0.031	3.9962
2484	158363	1598	22	5.81	0.017	3.9930
2484	157574	1590		5.87	0.017	3.9922
2484	153896	1549		6.15	0.018	3.9824
873	60516	508	40	2.40	0.007	3.9840
873	52641	420		2.98	0.009	3.9846
873	60052	503		2.44	0.007	3.9804
2426	148582	1489	12	6.16	0.018	3.9827
2426	151106	1518		5.98	0.017	3.9801
2426	150273	1508		6.02	0.017	3.9962

Table S 10: Data obtained for the solubility experiments using CO_2 in 4.8 ml sulfolane where initial pressure (p_{ini}) is expressed in mbar, peak area is expressed in arbitrary units (a.u), p_{eq} is expressed in mbar, the repeatability (RPT) of these triplicate pressures is expressed in mbar, the gas capacity (C_{gas}) is expressed in mg/g, x_{gas} is the mole fraction of gas in the solvent and the mass of sorbent (m_{sorb}) is expressed in g.

CO_2						
Sulfolane						
p_{ini} (mbar)	Peak area (a.u.)	p_{eq} (mbar)	RPT. p_{eq} (mbar)	C_{gas} (mg/g)	x_{gas}	m_{sorb} (g)
2799	150184	1507	3	5.60	0.015	6.0428
2799	149830	1503		5.62	0.015	6.0430
2799	149545	1500		5.63	0.015	6.0407
793	48330	372	3	1.82	0.005	6.0447
793	47862	367		1.85	0.005	6.0411
793	47616	364		1.85	0.005	6.0664
3516	177323	1810	8	7.40	0.020	6.0449
3516	178033	1818		7.36	0.020	6.0456
3516	176303	1798		7.45	0.020	6.0442
2180	119738	1168	2	4.39	0.012	6.0420
2180	120112	1172		4.36	0.012	6.0558
2180	119648	1167		4.39	0.012	6.0491
1646	106337	1019	7	2.72	0.007	6.0428
1646	105163	1006		2.78	0.008	6.0430
1646	104860	1002		2.79	0.008	6.0407
433	33623	208	10	0.97	0.003	6.0447
433	34497	218		0.93	0.003	6.0411
433	32374	194		1.03	0.003	6.0664
1285	85119	782	4	2.18	0.006	6.0449
1285	85522	787		2.16	0.006	6.0456
1285	84569	776		2.21	0.006	6.0442
1927	120520	1177	10	3.25	0.009	6.0420
1927	119220	1162		3.31	0.009	6.0558
1927	118383	1153		3.35	0.009	6.0491

Table S 11: Data obtained for the solubility experiments using CO_2 in 4.8 ml DMSO where initial pressure (p_{ini}) is expressed in mbar, peak area is expressed in arbitrary units (a.u), p_{eq} is expressed in mbar, the repeatability (RPT) of these triplicate pressures is expressed in mbar, the gas capacity (C_{gas}) is expressed in mg/g, x_{gas} is the mole fraction of gas in the solvent and the mass of sorbent (m_{sorb}) is expressed in g.

CO_2						
DMSO						
p_{ini} (mbar)	Peak area (a.u.)	p_{eq} (mbar)	RPT. p_{eq} (mbar)	C_{gas} (mg/g)	x_{gas}	m_{sorb} (g)
1904	99651	944	4	4.77	0.008	5.2785
1904	99001	937		4.80	0.008	5.2776
1904	99920	947		4.75	0.008	5.2787
987	50707	399	3	2.92	0.005	5.2866
987	50871	400		2.91	0.005	5.2768
987	50152	392		2.95	0.005	5.2880
3169	152324	1531	8	8.12	0.014	5.2823
3169	153959	1549		8.04	0.014	5.2817
3169	152667	1535		8.11	0.014	5.2777
2258	116498	1132	6	5.58	0.010	5.2906
2258	115333	1119		5.65	0.010	5.2826
2258	115348	1119		5.66	0.010	5.2765
681	42025	302	1	1.88	0.003	5.2785
681	41886	300		1.89	0.003	5.2776
681	41743	299		1.90	0.003	5.2787
1760	100449	953	6	4.00	0.007	5.2866
1760	100159	950		4.02	0.007	5.2768
1760	101436	964		3.94	0.007	5.2880
1130	67559	586	3	2.70	0.005	5.2823
1130	67151	582		2.72	0.005	5.2817
1130	67865	590		2.68	0.005	5.2777
2254	126130	1239	2	5.03	0.009	5.2826
2254	125751	1235		5.06	0.009	5.2765

Table S 12: Data obtained for the solubility experiments using CO_2 in 4.8 ml PEG200 where initial pressure (p_{ini}) is expressed in mbar, peak area is expressed in arbitrary units (a.u), p_{eq} is expressed in mbar, the repeatability (RPT) of these triplicate pressures is expressed in mbar, the gas capacity (C_{gas}) is expressed in mg/g, x_{gas} is the mole fraction of gas in the solvent and the mass of sorbent (m_{sorb}) is expressed in g.

CO_2						
PEG200						
p_{ini} (mbar)	Peak area (a.u.)	p_{eq} (mbar)	RPT. p_{eq} (mbar)	C_{gas} (mg/g)	x_{gas}	m_{sorb} (g)
1637	112831	1091	4	2.65	0.012	5.4049
1637	111993	1082		2.70	0.012	5.3872
1637	111997	1082		2.69	0.012	5.4062
663	48806	377	1	1.39	0.006	5.3891
663	49055	380		1.37	0.006	5.4014
663	49079	380		1.37	0.006	5.3920
2714	168681	1714	3	4.88	0.022	5.3777
2714	168008	1706		4.89	0.022	5.4001
2714	168308	1709		4.86	0.022	5.4169
814	64888	557	3	1.25	0.006	5.4049
814	65486	563		1.22	0.006	5.3872
814	64946	557		1.24	0.006	5.4062
1383	105335	1007	7	1.83	0.008	5.3891
1383	104702	1000		1.86	0.008	5.4014
1383	106249	1018		1.78	0.008	5.3920
1761	128613	1267	13	2.41	0.011	5.3777
1761	128761	1269		2.39	0.011	5.4001
1761	131223	1296		2.25	0.010	5.4169

Table S 13: Data obtained for the solubility experiments using CO_2 in 4.8 ml [C_6C_1Im][NTf_2] where initial pressure (p_{ini}) is expressed in mbar, peak area is expressed in arbitrary units (a.u), p_{eq} is expressed in mbar, the repeatability (RPT) of these triplicate pressures is expressed in mbar, the gas capacity (C_{gas}) is expressed in mg/g, x_{gas} is the mole fraction of gas in the solvent and the mass of sorbent (m_{sorb}) is expressed in g.

CO_2						
[C_6C_1Im][NTf_2]						
p_{ini} (mbar)	Peak area (a.u.)	p_{eq} (mbar)	RPT. p_{eq} (mbar)	C_{gas} (mg/g)	x_{gas}	m_{sorb} (g)
1480	86734	800		2.69	0.027	6.6142
1480	86115	793	9	2.72	0.027	6.6175
1480	84844	779		2.77	0.027	6.6229
1927	96743	912		4.01	0.039	6.6290
1927	98434	931	46	3.94	0.038	6.6320
1927	106272	1018		3.62	0.035	6.5890
570	33051	202		1.46	0.015	6.6004
570	33320	205	4	1.45	0.015	6.6105
570	32437	195		1.48	0.015	6.6269
2444	128871	1270	7	4.66	0.045	6.5987
2444	130214	1285		4.60	0.045	6.6101
1860	105121	1005		3.39	0.033	6.6142
1860	105406	1008	3	3.37	0.033	6.6175
1860	105683	1011		3.36	0.033	6.6229
849	51625	409		1.74	0.017	6.6290
849	51349	406	2	1.75	0.017	6.6320
849	51196	404		1.77	0.018	6.5890
2181	120732	1179		3.98	0.039	6.6004
2181	118161	1150	13	4.09	0.040	6.6105
2181	120366	1175		3.98	0.039	6.6269
1064	64133	548		2.04	0.020	6.6411

Table S 14: Fits of the data used obtained using the data in tables above relating to physisorbant materials where x is pressure and y is mole fraction. Fits are calculated using 5 ml calibration curves and the equilibrated material data relating to individual sorbents using calculations described in the experimental section. M is the gradient of the fit, c is the y intercept of the fit, S.D. x_{gas} is the average standard deviation of the mole fraction from the fit in the Y estimate and R^2 is used as a measure of the variance of the data to the fit.

Material	m	c	av. S.D. x_{gas}	R^2
Glycerol	0.0027	-0.00087	0.000364	0.960
Ethylene glycol	0.0033	-0.00075	0.000946	0.841
Octanol	0.0121	-0.00034	0.002108	0.952
Sulfolane	0.0100	-0.00046	0.001884	0.906
DMSO	0.0076	0.00095	0.001151	0.904
PEG 200	0.0110	-0.00035	0.002859	0.813
$[C_6C_1Im][NTf_2]$	0.0272	0.00718	0.001797	0.973

Table S 15: Data obtained for the solubility experiments using CO_2 in 0.8 ml 30% MEA solution where initial pressure (p_{ini}) is expressed in mbar, peak area is expressed in arbitrary units (a.u), p_{eq} is expressed in mbar, the repeatability (RPT) of these triplicate pressures is expressed in mbar, the gas capacity (C_{gas}) is expressed in mg/g, and the mass of sorbent (m_{sorb}) is expressed in g.

CO_2					
MEA 30%					
p_{ini} (mbar)	Peak area (a.u.)	p_{eq} (mbar)	RPT. p_{eq} (mbar)	C_{gas} (mg/g)	m_{sorb} (g)
4520	76599	771	185	122.94	1.0121
4520	103873	1128		111.55	1.0093
4520	71683	707		125.24	1.0105
4993	99965	1077	50	128.75	1.0096
4993	98243	1054		129.50	1.0095
4993	107113	1170		125.38	1.0120
4709	96068	1026	55	120.35	1.0158
4709	89828	944		123.06	1.0154
4709	85874	893		124.80	1.0150
3552	20130	33	17	115.46	1.0116
3552	18984	18		115.79	1.0130
3552	22103	59		114.62	1.0115
2497	78	-229	0	89.40	1.0121
2497	59	-229		89.66	1.0093
2497	27	-230		89.56	1.0105
3966	37346	258	52	121.90	1.0096
3966	33732	211		123.46	1.0095
3966	43416	338		119.01	1.0120

Table S 16: Data obtained for the solubility experiments using CO_2 in 3 Å molecular sieves where initial pressure (p_{ini}) is expressed in mbar, peak area is expressed in arbitrary units (a.u), p_{eq} is expressed in mbar, the repeatability (RPT) of these triplicate pressures is expressed in mbar, the gas capacity (C_{gas}) is expressed in mg/g, and the mass of sorbent (m_{sorb}) is expressed in g.

CO_2					
3 Å molecular sieves					
p_{ini} (mbar)	Peak area (a.u.)	p_{eq} (mbar)	RPT. p_{eq} (mbar)	C_{gas} (mg/g)	m_{sorb} (g)
1213	55812	477	36	50.76	0.4942
1213	61176	543		46.30	0.4927
1213	54586	461		50.34	0.5086
1865	103341	1067	14	53.28	0.5101
1865	105411	1093		53.39	0.4926
1865	102765	1060		54.67	0.5016
2883	181153	2034	17	58.38	0.4953
2883	181310	2036		57.52	0.5016
2883	178413	2000		59.62	0.5045
2145	128581	1381	15	51.72	0.5033
2145	125687	1345		54.66	0.4986
2145	127593	1369		52.93	0.4997
821	31554	0	103	56.59	0.4942
821	37289	246		39.72	0.4927
821	31459	174		43.33	0.5086
2296	140113	1524	20	51.54	0.5101
2296	143472	1566		50.49	0.4926
2296	140154	1525		52.38	0.5016
1720	102012	1051	13	46.03	0.4953
1720	101107	1039		46.22	0.5016
1720	99559	1020		47.25	0.5045

Table S 17: Data obtained for the solubility experiments using CO_2 in 4 Å molecular sieves where initial pressure (p_{ini}) is expressed in mbar, peak area is expressed in arbitrary units (a.u), p_{eq} is expressed in mbar, the repeatability (RPT.) of these triplicate pressures is expressed in mbar, the gas capacity (C_{gas}) is expressed in mg/g, and the mass of sorbent (m_{sorb}) is expressed in g.

CO_2					
4 Å molecular sieves					
p_{ini} (mbar)	Peak area (a.u.)	p_{eq} (mbar)	RPT. p_{eq} (mbar)	C_{gas} (mg/g)	m_{sorb} (g)
1803	144309	1442	25	61.39	0.2004
1803	142266	1419		62.13	0.2105
1803	138906	1382		71.52	0.2007
1962	149074	1495	23	89.33	0.1781
1962	149862	1504		71.48	0.2184
1962	145182	1452		99.02	0.1756
2085	149492	1500	62	84.71	0.2354
2085	160562	1623		90.45	0.1740
691	24588	107	15	105.97	0.1876
691	21884	77		102.14	0.2047
691	21716	75		99.16	0.2115
Using different calibration curve with 20 G needles					
718	36946	245	27	80.38	0.2004
718	36798	243		76.79	0.2105
718	31740	187		90.11	0.2007
2275	158884	1604	65	128.28	0.1781
2275	152644	1535		115.46	0.2184
2275	166894	1694		112.79	0.1756
1862	121319	1186	14	97.88	0.2354
1862	123899	1214		104.31	0.2115
2534	162227	1642	37	162.05	0.1876
2534	169338	1721		135.32	0.2047
2534	169379	1721		130.90	0.2115

Table S 18: Data obtained for the solubility experiments using CO_2 in zeolite RHO where initial pressure (p_{ini}) is expressed in mbar, peak area is expressed in arbitrary units (a.u), p_{eq} is expressed in mbar, the repeatability (RPT.) of these triplicate pressures is expressed in mbar, the gas capacity (C_{gas}) is expressed in mg/g, and the mass of sorbent (m_{sorb}) is expressed in g.

CO_2					
Zeolite rho solid					
p_{ini} (mbar)	Peak area (a.u.)	p_{eq} (mbar)	RPT. p_{eq} (mbar)	C_{gas} (mg/g)	m_{sorb} (g)
2651	144819	1583	23	120.23	0.3027
2651	140204	1525		131.60	0.2914
2651	142220	1550		125.27	0.2993
3332	177087	1984	140	146.94	0.3126
3332	175306	1961		155.11	0.301
3332	152350	1676		166.73	0.3383
781	32849	191	12	63.01	0.3188
781	35166	220		63.67	0.3001
781	33332	197		61.64	0.3226
1845	92604	934	10	97.36	0.3188
1845	91114	915		104.52	0.303
1845	90853	912		105.41	0.3015

Table S 19: Data obtained for the solubility experiments using CO_2 with 0.8 ml zeolite RHO 25 wt% in genosorb where initial pressure (p_{ini}) is expressed in mbar, peak area is expressed in arbitrary units (a.u), p_{eq} is expressed in mbar, the gas capacity (C_{gas}) is expressed in mg/g, and the mass of sorbent (m_{sorb}) is expressed in g.

CO_2				
Zeolite rho 25 wt% in genosorb® 1753				
p_{ini} (mbar)	Peak area (a.u.)	p_{eq} (mbar)	C_{gas} (mg/g)	m_{sorb} (g)
2345	121016	1182	33.22	1.16
2345	121241	1185	32.74	1.18
2345	118447	1154	33.35	1.19
3043	151559	1523	42.56	1.19
3043	157026	1584	40.93	1.18
1674	74883	668	28.05	1.19
1674	84866	779	27.01	1.10

Table S 20: Fits of the data used obtained using the data in tables above relating to high capacity materials where x is pressure and y is mole fraction. Fits are calculated using empty vial calibration curves and the equilibrated material data relating to individual sorbents using calculations described in the experimental section. Where M is the gradient of the fit, c is the y intercept of the fit, S.D. $mg_{\text{gas}}/g_{\text{sorbent}}$ is the average standard deviation of the capacity from the fit in the Y estimate and R^2 is used as a measure of the variance of the data to the fit.

Material	m	c	av. S.D. $mg_{\text{gas}}/g_{\text{sorbent}}$	R^2
MEA 30%	0.0085	116.95	2.93	0.635
Zeolite rho solid	0.0166	14.86	1.80	0.922
Zeolite rho 25 wt% in genosorb® 1753	0.0480	54.56	5.60	0.974
4 Å molecular sieves	0.0337	72.40	14.89	0.700

Table S 21: Data obtained for the solubility experiments using CH_4 in 4.8 ml PEG200 where initial pressure (p_{ini}) is expressed in mbar, peak area is expressed in arbitrary units (a.u), p_{eq} is expressed in mbar, the repeatability (RPT) of these triplicate pressures is expressed in mbar, the gas capacity (C_{gas}) is expressed in mg/g, x_{gas} is the mole fraction of gas in the solvent and the mass of sorbent (m_{sorb}) is expressed in g.

CH_4						
PEG-200						
p_{ini} (mbar)	Peak area (a.u.)	p_{eq} (mbar)	RPT. p_{eq} (mbar)	C_{gas} (mg/g)	x_{gas}	m_{sorb} (g)
977	137372	985	1	-0.01	-0.0002	5.405
977	137554	986		-0.02	-0.0002	5.387
977	137308	984		-0.01	-0.0002	5.406
1758	223062	1700	26	0.10	0.0013	5.389
1758	229012	1749		0.02	0.0002	5.401
1758	230100	1759		0.00	0.0000	5.407
2184	279001	2166	9	0.03	0.0004	5.405
2184	276955	2149		0.06	0.0008	5.387
919	129734	921	1	0.00	0.0000	5.389
919	129883	922		-0.01	-0.0001	5.401
919	129641	920		0.00	0.0000	5.3777
1510	198018	1491	41	0.03	0.0004	5.4001
1510	203526	1537		-0.05	-0.0006	5.4169
1510	191526	1437		0.13	0.0016	5.4069

Table S 22: Data obtained for the solubility experiments using CH_4 in 4.8 ml DMSO where initial pressure (p_{ini}) is expressed in mbar, peak area is expressed in arbitrary units (a.u), p_{eq} is expressed in mbar, the repeatability (RPT.) of these triplicate pressures is expressed in mbar, the gas capacity (C_{gas}) is expressed in mg/g, x_{gas} is the mole fraction of gas in the solvent and the mass of sorbent (m_{sorb}) is expressed in g.

CH_4						
DMSO						
p_{ini} (mbar)	Peak area (a.u.)	p_{eq} (mbar)	RPT. p_{eq} (mbar)	C_{gas} (mg/g)	x_{gas}	m_{sorb} (g)
2023	244398	1878	39	0.26	0.0013	5.2866
2023	240040	1841		0.33	0.0016	5.2768
2023	251276	1935		0.16	0.0008	5.2880
1240	159401	1169	10	0.13	0.0006	5.2823
1240	160790	1180		0.11	0.0005	5.2817
1240	162244	1192		0.09	0.0004	5.2777
787	108448	744	1	0.08	0.0004	5.2906
787	108256	742		0.08	0.0004	5.2826
787	108156	741		0.08	0.0004	5.2765
2558	294226	2293	43	0.48	0.0023	5.2866
2558	302373	2361		0.35	0.0017	5.2768
2558	306806	2398		0.29	0.0014	5.2880
757	105975	723	1	0.06	0.0003	5.2823
757	105721	721		0.07	0.0003	5.2817
757	105715	721		0.07	0.0003	5.2777
1734	215440	1636	20	0.18	0.0009	5.2906
1734	219496	1670		0.12	0.0006	5.2826
1734	213707	1622		0.20	0.0010	5.2765

Table S 23: Data obtained for the solubility experiments using CH_4 in 4.8 ml glycerol where initial pressure (p_{ini}) is expressed in mbar, peak area is expressed in arbitrary units (a.u), p_{eq} is expressed in mbar, the repeatability (RPT.) of these triplicate pressures is expressed in mbar, the gas capacity (C_{gas}) is expressed in mg/g, x_{gas} is the mole fraction of gas in the solvent and the mass of sorbent (m_{sorb}) is expressed in g.

CH_4						
Glycerol						
p_{ini} (mbar)	Peak area (a.u.)	p_{eq} (mbar)	RPT. p_{eq} (mbar)	C_{gas} (mg/g)	x_{gas}	m_{sorb} (g)
1618	218578	1662	97	-0.070	-0.00040	6.0122
1618	219284	1668		-0.080	-0.00046	6.0178
1618	194181	1459		0.253	0.00145	5.9907
1859	246727	1897	117	-0.061	-0.00035	6.0099
1859	247246	1902		-0.068	-0.00039	5.9906
1859	217307	1652		0.328	0.00188	6.0191
786	112038	774	1	0.020	0.00011	6.0123
786	111889	772		0.022	0.00012	5.9979
786	112143	774		0.018	0.00011	6.0101
2440	307117	2401	13	0.062	0.00036	6.0071
2440	304299	2378		0.099	0.00057	6.0072
2440	303671	2372		0.108	0.00062	5.9898
607	84731	546	0	0.097	0.00056	6.0300
607	84708	546		0.098	0.00056	5.9935
607	84687	545		0.098	0.00056	6.0037
2108	273836	2123	10	-0.024	-0.00014	5.9967
2108	274900	2132		-0.039	-0.00022	5.9959
2108	272115	2109		-0.002	-0.00001	6.0095
1453	195803	1472	19	-0.031	-0.00018	6.0003
1453	199085	1500		-0.074	-0.00043	6.0184
1454	193483	1453	25	0.002	0.00001	6.0000
1454	199732	1505		-0.081	-0.00047	6.0146
1454	199742	1505		-0.081	-0.00047	6.0116
814	118328	826	2	-0.019	-0.00011	6.0300
814	118860	830		-0.026	-0.00015	5.9935
814	118784	830		-0.025	-0.00014	6.0037
2542	319498	2504	12	0.060	0.00034	5.9967
2542	316059	2476		0.105	0.00061	5.9959
2542	317381	2487		0.088	0.00050	6.0095

CH_4						
Glycerol						
p_{ini} (mbar)	Peak area (a.u.)	p_{eq} (mbar)	RPT. p_{eq} (mbar)	C_{gas} (mg/g)	x_{gas}	m_{sorb} (g)
1383	193837	1456	11	-0.116	-0.00067	6.0003
1383	191050	1433		-0.079	-0.00045	5.9980
1383	193820	1456		-0.115	-0.00066	6.0184
2032	267143	2068	10	-0.056	-0.00032	6.0000
2032	268364	2078		-0.072	-0.00042	6.0146
2032	270018	2092		-0.094	-0.00054	6.0116
1714	223728	1705	72	0.014	0.00008	6.0122
1714	234156	1792		-0.124	-0.00071	6.0178
1714	213143	1617		0.154	0.00089	5.9907
2600	311161	2435	24	0.262	0.00151	6.0099
2600	315158	2468		0.210	0.00121	5.9906
1349	190437	1428	14	-0.125	-0.00072	6.0071
1349	193088	1450		-0.160	-0.00092	6.0072
1349	189002	1416		-0.106	-0.00061	5.9898

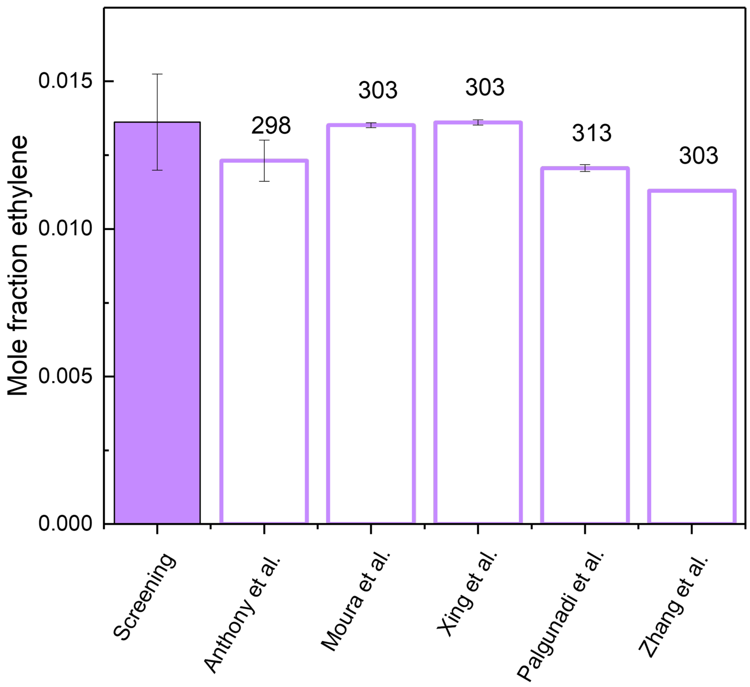

Figure S 6: C_2H_4 sorption results, in gas mole fraction, obtained from HS-GC method at 308 K (solid bars) and those published in the literature at 303 K (striped bars), at 1000 mbar, for $[C_4C_1Im][NTf_2]$.[3–7]. Measurements/calibrations were obtained using 3 mL of vial occupied volume. Error bars represent the the average absolute deviation of the data to the fit

.

Table S 24: Data obtained for the solubility experiments using CH_4 in 4.8 ml 1-octanol where initial pressure (p_{ini}) is expressed in mbar, peak area is expressed in arbitrary units (a.u), p_{eq} is expressed in mbar, the repeatability (RPT) of these triplicate pressures is expressed in mbar, the gas capacity (C_{gas}) is expressed in mg/g, x_{gas} is the mole fraction of gas in the solvent and the mass of sorbent (m_{sorb}) is expressed in g.

CH_4						
1-octanol						
p_{ini} (mbar)	Peak area (a.u.)	p_{eq} (mbar)	RPT. p_{eq} (mbar)	C_{gas} (mg/g)	x_{gas}	m_{sorb} (g)
1881	222488	1695	21	0.45	0.0036	3.9735
1881	222057	1691		0.45	0.0037	3.9800
1881	227498	1737		0.34	0.0028	3.9823
2380	273703	2122	29	0.62	0.0050	3.9833
2380	265888	2057		0.77	0.0062	3.9780
2380	267103	2067		0.75	0.0061	3.9849
566	71607	436	0	0.31	0.0025	3.9766
566	71528	436		0.31	0.0025	3.9816
566	71579	436		0.31	0.0025	3.9820
1802	217655	1655	11	0.35	0.0029	3.9800
1802	220256	1676		0.30	0.0024	3.9823
1476	183327	1368	18	0.26	0.0021	3.9865
1476	184383	1377		0.24	0.0019	3.9804
1476	179356	1335		0.34	0.0027	3.9840
940	122435	860	9	0.19	0.0015	3.9833
940	120494	844		0.23	0.0019	3.9780
940	122955	865		0.18	0.0015	3.9849
2234	259492	2004	20	0.55	0.0045	3.9766
2234	264636	2047		0.45	0.0036	3.9816
2234	259824	2006		0.54	0.0044	3.9820

Table S 25: Data obtained for the solubility experiments using CH_4 in 5 ml ethylene glycol where initial pressure (p_{ini}) is expressed in mbar, peak area is expressed in arbitrary units (a.u), p_{eq} is expressed in mbar, the repeatability (RPT) of these triplicate pressures is expressed in mbar, the gas capacity (C_{gas}) is expressed in mg/g, x_{gas} is the mole fraction of gas in the solvent and the mass of sorbent (m_{sorb}) is expressed in g.

CH_4						
ethylene glycol						
p_{ini} (mbar)	Peak area (a.u.)	p_{eq} (mbar)	RPT. p_{eq} (mbar)	C_{gas} (mg/g)	x_{gas}	m_{sorb} (g)
1873	243838	1873	3	0.000	-0.000001	5.3370
1873	244564	1879		-0.011	-0.000043	5.3446
1873	243773	1873		0.001	0.000003	5.3455
931	129822	922	0	0.016	0.000063	5.3337
931	129885	922		0.015	0.000059	5.3527
931	129740	921		0.017	0.000067	5.3439
1540	207866	1573	11	-0.059	-0.000229	5.3374
1540	204786	1547		-0.013	-0.000051	5.3351
1540	206521	1562		-0.039	-0.000151	5.3351
1608	216062	1641	9	-0.060	-0.000231	5.3442
1608	214206	1626		-0.032	-0.000124	5.3366
1608	213515	1620		-0.022	-0.000084	5.3492
2820	333451	2621	66	0.356	0.001378	5.3370
2820	317409	2487		0.594	0.002298	5.3446
2820	335068	2634		0.331	0.001283	5.3455
1186	163646	1204	10	-0.032	-0.000126	5.3337
1186	160661	1179		0.012	0.000047	5.3527
1186	162150	1192		-0.010	-0.000039	5.3439
803	114836	797	1	0.011	0.000042	5.3374
803	115131	799		0.006	0.000025	5.3351
803	115193	800		0.006	0.000021	5.3351
1973	257601	1988	27	-0.027	-0.000103	5.3442
1973	259214	2001		-0.051	-0.000197	5.3366
1973	251646	1938		0.062	0.000240	5.3492
712	100998	681	1	0.054	0.000210	5.3603
712	101178	683		0.052	0.000201	5.3450
712	100974	681		0.055	0.000212	5.3528
2257	263612	2038	87	0.390	0.001509	5.3538
2257	284574	2213		0.078	0.000304	5.3485
1339	179371	1335	63	0.007	0.000026	5.3432
1339	181040	1349		-0.018	-0.000071	5.3399
1339	164302	1210		0.231	0.000894	5.3446
1829	237496	1820	11	0.016	0.000061	5.3603
1829	237390	1819		0.017	0.000067	5.3450
1829	240145	1842		-0.024	-0.000092	5.3528

Table S 26: Data obtained for the solubility experiments using CH_4 in 4.8 ml sulfolane where initial pressure (p_{ini}) is expressed in mbar, peak area is expressed in arbitrary units (a.u), p_{eq} is expressed in mbar, the repeatability (RPT) of these triplicate pressures is expressed in mbar, the gas capacity (C_{gas}) is expressed in mg/g, x_{gas} is the mole fraction of gas in the solvent and the mass of sorbent (m_{sorb}) is expressed in g.

CH_4						
Sulfolane						
p_{ini} (mbar)	Peak area (a.u.)	p_{eq} (mbar)	RPT. p_{eq} (mbar)	C_{gas} (mg/g)	x_{gas}	m_{sorb} (g)
1918	238958	1832	40	0.135	0.00101	6.0428
1918	246797	1898		0.032	0.00024	6.0430
1918	250456	1928		-0.016	-0.00012	6.0407
1136	154828	1131	10	0.009	0.00006	6.0447
1136	152394	1110		0.041	0.00030	6.0411
1136	155031	1132		0.006	0.00004	6.0664
2462	287650	2239	42	0.352	0.00264	6.0449
2462	295520	2304		0.249	0.00186	6.0456
2462	299805	2340		0.192	0.00144	6.0442
695	96746	646	0	0.077	0.00058	6.0420
695	96647	645		0.078	0.00059	6.0558
695	96636	645		0.079	0.00059	6.0491
2570	315389	2470	8	0.158	0.00118	6.0428
2570	317231	2485		0.133	0.00100	6.0407
1156	154339	1126	12	0.047	0.00035	6.0411
1156	157217	1150		0.009	0.00007	6.0664
496	67894	405	1	0.143	0.00107	6.0449
496	67864	405		0.143	0.00107	6.0456
496	67558	403		0.147	0.00111	6.0442
1968	254739	1964	17	0.006	0.00005	6.0420
1968	250697	1930		0.059	0.00044	6.0558
1968	255412	1970		-0.003	-0.00002	6.0491

Table S 27: Data obtained for the solubility experiments using CH_4 in 9.8 ml ethylene glycol where initial pressure (p_{ini}) is expressed in mbar, peak area is expressed in arbitrary units (a.u), p_{eq} is expressed in mbar, the repeatability (RPT) of these triplicate pressures is expressed in mbar, the gas capacity (C_{gas}) is expressed in mg/g, x_{gas} is the mole fraction of gas in the solvent and the mass of sorbent (m_{sorb}) is expressed in g.

CH_4						
ethylene glycol						
p_{ini} (mbar)	Peak area (a.u.)	p_{eq} (mbar)	RPT. p_{eq} (mbar)	C_{gas} (mg/g)	x_{gas}	m_{sorb} (g)
1622	178333	1351	12	0.16	0.0006	10.6724
1622	175262	1325		0.18	0.0007	10.6674
1622	178212	1350		0.16	0.0006	10.6708
2734	269753	2124	6	0.36	0.0014	10.7038
2734	271059	2135		0.36	0.0014	10.6409
878	100495	692	20	0.11	0.0004	10.6582
878	95759	652		0.13	0.0005	10.6524
2313	238293	1858	41	0.27	0.0010	10.7088
2313	244747	1913		0.24	0.0009	10.6482
2313	232870	1812		0.30	0.0012	10.6675

Table S 28: Data obtained for the solubility experiments using CH_4 in 9.8 ml ethylene glycol where initial pressure (p_{ini}) is expressed in mbar, peak area is expressed in arbitrary units (a.u), p_{eq} is expressed in mbar, the repeatability (RPT) of these triplicate pressures is expressed in mbar, the gas capacity (C_{gas}) is expressed in mg/g, x_{gas} is the mole fraction of gas in the solvent and the mass of sorbent (m_{sorb}) is expressed in g.

CH_4						
$[C_6C_1Im][NTf_2]$						
p_{ini} (mbar)	Peak area (a.u.)	p_{eq} (mbar)	RPT. p_{eq} (mbar)	C_{gas} (mg/g)	x_{gas}	m_{sorb} (g)
2519	251965	2263	38	0.12	0.0034	13.4993
2519	258927	2330		0.09	0.0025	13.4610
2519	261385	2354		0.08	0.0022	13.4912
2014	214677	1904	75	0.05	0.0014	13.4990
2014	203030	1792		0.10	0.0029	13.5204
2014	195817	1723		0.13	0.0038	13.7470
1448	143725	1221	58	0.11	0.0030	13.4490
1448	131715	1105		0.16	0.0045	13.4969

Table S 29: Data obtained for the solubility experiments using CH_4 in 9.8 ml octanol where initial pressure (p_{ini}) is expressed in mbar, peak area is expressed in arbitrary units (a.u), p_{eq} is expressed in mbar, the repeatability (RPT) of these triplicate pressures is expressed in mbar, the gas capacity (C_{gas}) is expressed in mg/g, x_{gas} is the mole fraction of gas in the solvent and the mass of sorbent (m_{sorb}) is expressed in g.

CH_4						
1-octanol						
p_{ini} (mbar)	Peak area (a.u.)	p_{eq} (mbar)	RPT. p_{eq} (mbar)	C_{gas} (mg/g)	x_{gas}	m_{sorb} (g)
2588	218487	1941	50	0.51	0.0041	8.0843
2588	210266	1862		0.57	0.0046	8.1550
2588	222673	1981		0.47	0.0038	8.2762
2092	187662	1644	33	0.35	0.0028	8.1128
2092	194903	1714		0.30	0.0024	8.1223
2092	194903	1714		0.29	0.0024	8.1803
1763	167354	1448	10	0.24	0.0020	8.1650
1763	167321	1448		0.25	0.0020	8.1330
1763	169498	1469		0.23	0.0019	8.1912
1233	127500	1064	88	0.13	0.0011	8.1549
1233	123917	1030		0.16	0.0013	8.1024
1233	144819	1231		0.00	0.0000	8.1634
2271	186749	1635	0	0.50	0.0040	8.1550
2271	186732	1635		0.49	0.0040	8.2762
1087	98653	787	4	0.24	0.0019	8.1128
1087	97799	778		0.24	0.0020	8.1223
1087	97807	778		0.24	0.0019	8.1803
1873	157827	1357	30	0.40	0.0033	8.1650
1873	150160	1283		0.46	0.0037	8.1330
1873	153585	1316		0.43	0.0035	8.1912
2911	235135	2101	15	0.63	0.0051	8.1549
2911	233182	2082		0.65	0.0053	8.1024
2911	231416	2065		0.66	0.0053	8.1634

Table S 30: Data obtained for the solubility experiments using CH_4 in 9.8 ml DMSO where initial pressure (p_{ini}) is expressed in mbar, peak area is expressed in arbitrary units (a.u), p_{eq} is expressed in mbar, the repeatability (RPT) of these triplicate pressures is expressed in mbar, the gas capacity (C_{gas}) is expressed in mg/g, x_{gas} is the mole fraction of gas in the solvent and the mass of sorbent (m_{sorb}) is expressed in g.

CH_4						
DMSO						
p_{ini} (mbar)	Peak area (a.u.)	p_{eq} (mbar)	RPT. p_{eq} (mbar)	C_{gas} (mg/g)	x_{gas}	m_{sorb} (g)
2952	263861	2074	12	0.51	0.0025	10.97
2952	262973	2067		0.51	0.0025	10.97
2952	266314	2095		0.50	0.0024	10.99
1033	127699	922	3	0.06	0.0003	10.98
1033	126939	916		0.07	0.0003	10.98
1033	127236	918		0.07	0.0003	10.99
2276	233494	1817	38	0.27	0.0013	10.98
2276	242425	1893		0.22	0.0011	10.98
822	100699	694	36	0.07	0.0004	10.97
822	92138	621		0.12	0.0006	10.98
822	101535	701		0.07	0.0003	10.98

Table S 31: Data obtained for the solubility experiments using C_2H_4 in 2.8 ml $[C_4C_1Im][NTf_2]$ + $Ag[NTf_2]$ where initial pressure (p_{ini}) is expressed in mbar and peak area is expressed in arbitrary units (a.u), p_{eq} is expressed in mbar, the repeatability (RPT) of these triplicate pressures is expressed in mbar, the gas capacity (C_{gas}) is expressed in mg/g, x_{gas} is the mole fraction of gas in the solvent and the mass of sorbent (m_{sorb}) is expressed in g.

C_2H_4						
$[C_4C_1Im][NTf_2]$ + $Ag[NTf_2]$						
p_{ini} (mbar)	Peak area (a.u.)	p_{eq} (mbar)	RPT. p_{eq} (mbar)	C_{gas} (mg/g)	x_{gas}	m_{sorb} (g)
794	73935	283	6	1.93	0.0299	4.7167
794	76935	293		1.93	0.0299	4.6176
794	73935	283		1.95	0.0302	4.6672
1212	127141	459	57	2.84	0.0434	4.7149
1212	131106	472		2.79	0.0426	4.7258
1212	158677	563		2.45	0.0376	4.7204
1404	155361	552	6	3.20	0.0486	4.7430
1404	155361	552		3.22	0.0490	4.7023
1404	158677	563		3.17	0.0482	4.7227
2067	249936	865	26	4.54	0.0676	4.7167
2067	265421	916		4.44	0.0662	4.6176
2067	257679	890		4.49	0.0669	4.6672
2928	403394	1372	38	5.88	0.0858	4.7149
2928	387283	1318		6.06	0.0883	4.7258
3720	580877	1958	22	6.62	0.0955	4.7430
3720	580877	1958		6.67	0.0963	4.7023
3720	569333	1920		6.79	0.0978	4.7227

Table S 32: Data obtained for the solubility experiments using C_2H_6 in 2.8 ml $[C_4C_1Im][NTf_2]$ + $Ag[NTf_2]$ where initial pressure (p_{ini}) is expressed in mbar and peak area is expressed in arbitrary units (a.u), p_{eq} is expressed in mbar, the repeatability (RPT) of these triplicate pressures is expressed in mbar, the gas capacity (C_{gas}) is expressed in mg/g, x_{gas} is the mole fraction of gas in the solvent and the mass of sorbent (m_{sorb}) is expressed in g.

C_2H_6						
$[C_4C_1Im][NTf_2]$ + $Ag[NTf_2]$						
p_{ini} (mbar)	Peak area (a.u.)	p_{eq} (mbar)	RPT. p_{eq} (mbar)	C_{gas} (mg/g)	x_{gas}	m_{sorb} (g)
800	195493	701	15	0.40	0.0059	4.7077
800	203484	731		0.28	0.0042	4.7267
800	199489	716		0.34	0.0050	4.7172
1256	305228	1103	8	0.62	0.0092	4.6997
1256	300651	1086		0.69	0.0102	4.7013
1256	302940	1094		0.66	0.0097	4.7005
1527	367682	1331	13	0.79	0.0128	4.7180
1527	362779	1313		0.87	0.0128	4.6934

Table S 33: Data obtained for the solubility experiments using C_2H_4 in 2.8 ml [C_4C_1Im][NTf_2] where initial pressure (p_{ini}) is expressed in mbar and peak area is expressed in arbitrary units (a.u), p_{eq} is expressed in mbar, the repeatability (RPT) of these triplicate pressures is expressed in mbar, the gas capacity (C_{gas}) is expressed in mg/g, x_{gas} is the mole fraction of gas in the solvent and the mass of sorbent (m_{sorb}) is expressed in g.

C_2H_4						
[C_4C_1Im][NTf_2]						
p_{ini} (mbar)	Peak area (a.u.)	p_{eq} (mbar)	RPT. p_{eq} (mbar)	C_{gas} (mg/g)	x_{gas}	m_{sorb} (g)
683	169332	564	4	0.55	0.0082	4.0419
683	167032	557		0.59	0.0087	4.0362
683	167848	559		0.58	0.0086	4.0218
909	232284	772	2	0.64	0.0094	4.0436
909	231959	771		0.64	0.0095	4.0348
909	231305	769		0.65	0.0097	4.0390
1171	298313	990	6	0.84	0.0124	4.0407
1171	299348	994		0.83	0.0122	4.0342
1171	295955	982		0.88	0.0130	4.0174
682	163965	547	1	0.63	0.0094	4.0419
682	164733	549		0.62	0.0092	4.0362
682	163931	546		0.64	0.0094	4.0218
928	231402	769	5	0.74	0.0109	4.0436
928	228409	759		0.79	0.0116	4.0348
928	229728	764		0.77	0.0113	4.0390
1169	294081	976	10	0.90	0.0133	4.0407
1169	299688	995		0.81	0.0120	4.0342
1169	294963	979		0.89	0.0131	4.0174

Table S 34: Data obtained for the solubility experiments using C_2H_6 in 2.8 ml $[C_4C_1Im][NTf_2]$ where initial pressure (p_{ini}) is expressed in mbar and peak area is expressed in arbitrary units (a.u), p_{eq} is expressed in mbar, the repeatability (RPT) of these triplicate pressures is expressed in mbar, the gas capacity (C_{gas}) is expressed in mg/g, x_{gas} is the mole fraction of gas in the solvent and the mass of sorbent (m_{sorb}) is expressed in g.

C_2H_6						
$[C_4C_1Im][NTf_2]$						
p_{ini} (mbar)	Peak area (a.u.)	p_{eq} (mbar)	RPT. p_{eq} (mbar)	C_{gas} (mg/g)	x_{gas}	m_{sorb} (g)
700	182907	608	2	0.46	0.0064	4.0419
700	181743	604		0.45	0.0066	4.0362
700	182932	608		0.43	0.0064	4.0218
971	254799	851	0	0.56	0.0083	4.0436
971	254957	851		0.56	0.0083	4.0348
971	254978	851		0.56	0.0083	4.0390
1300	340603	1140	7	0.74	0.0110	4.0407
1300	342053	1145		0.72	0.0106	4.0342
1300	338073	1132		0.79	0.0116	4.0174
804	211270	704	1	0.50	0.0069	4.0419
804	211495	705		0.46	0.0069	4.0362
804	210800	702		0.48	0.0071	4.0218
1029	271034	906	1	0.57	0.0085	4.0436
1029	271651	908		0.57	0.0084	4.0348
1029	270964	905		0.58	0.0085	4.0390
1311	357400	1197	3	0.53	0.0078	4.0407
1311	357283	1197		0.53	0.0079	4.0342
1311	356061	1193		0.55	0.0082	4.0174

Table S 35: Data obtained for the solubility experiments using 50/50 C_2H_6/C_2H_4 in 3 ml $[C_4C_1Im][NTf_2]$ + $Ag[NTf_2]$ where partial pressure pressure of each gas (p_{ini}) is expressed in mbar, peak area is expressed in arbitrary units (a.u), p_{eq} is expressed in mbar, the repeatability (RPT) of these triplicate pressures is expressed in mbar, the gas capacity (C_{gas}) is expressed in mg/g, x_{gas} is the mole fraction of gas in the solvent and the mass of sorbent (m_{sorb}) is expressed in g.

$[C_4C_1Im][NTf_2]$ + $Ag[NTf_2]$						
50/50 C_2H_6/C_2H_4 mixed gas data						
p_{ini} (mbar)	Peak area (a.u.)	p_{eq} (mbar)	RPT. p_{eq} (mbar)	C_{gas} (mg/g)	x_{gas}	m_{sorb} (g)
C_2H_4						
1145	124517	460	15	4.35	0.040	4.7077
1145	116603	430		4.52	0.041	4.7267
1145	120560	445		4.44	0.040	4.7172
1617	178905	664	2	6.07	0.055	4.6997
1617	177866	660		6.09	0.055	4.7013
1617	178385	662		6.08	0.055	4.7005
510	44128	159	0	2.23	0.021	4.7180
510	43973	158		2.24	0.021	4.6934
510	44051	158		2.24	0.021	4.7057
C_2H_6						
1145	292562	1090	36	0.22	0.0033	4.7077
1145	272886	1017		0.51	0.0076	4.7267
1145	282724	1054		0.37	0.0054	4.7172
1617	367302	1364	3	1.02	0.0150	4.6997
1617	365452	1357		1.05	0.0154	4.7013
1617	366377	1361		1.04	0.0152	4.7005
510	120892	459	0	0.20	0.0030	4.7180
510	121035	460		0.20	0.0030	4.6934
510	120963	460		0.20	0.0030	4.7057

Table S 36: Fits of the data used obtained using the data in tables above relating to physisorbant materials where x is pressure and y is mole fraction. Fits are calculated using 4.8 mL calibration curves and the equilibrated material data relating to individual sorbents using calculations described in the experimental section. Where M is the gradient of the fit, c is the y intercept of the fit , av. S.D. x_{gas} is the average standard deviation of the mole fraction from the fit and R^2 is used as a measure of the variance of the data to the fit.

Gas	Material	m	c	av. S.D. $mg_{\text{gas}}/g_{\text{sorbent}}$	R^2
C_2H_4	$[C_4C_1Im][NTf_2]$ + $Ag[NTf_2]$	5.43E-05	0.0160	0.00370	0.967
C_2H_6	$[C_4C_1Im][NTf_2]$ + $Ag[NTf_2]$	1.26E-05	-0.004	0.000717	0.961
C_2H_4	$[C_4C_1Im][NTf_2]$	94.5E-05	0.00291	0.000599	0.908
C_2H_6	$[C_4C_1Im][NTf_2]$	87E-05	0.00092	0.000333	0.963
50/50 C_2H_6	$[C_4C_1Im][NTf_2]$ + $Ag[NTf_2]$	1.21E-05	-0.00369	0.00327	0.712
50/50 C_2H_4	$[C_4C_1Im][NTf_2]$ + $Ag[NTf_2]$	6.71E-05	0.01031	0.000963	0.996

References

(1) Bonhote, P.; Dias, A.-P.; Papageorgiou, N.; Kalyanasundaram, K.; Grätzel, M. *Inorg. Chem.* **1996**, *35*, 1168–1178.

(2) Agel, F.; Pitsch, F.; Krull, F. F.; Schulz, P.; Wessling, M.; Melin, T.; Wasserscheid, P. *Phys. Chem. Chem. Phys.* **2011**, *13*, 725–731.

(3) Anthony, J. L.; Anderson, J. L.; Maginn, E. J.; Brennecke, J. F. *J. Phys. Chem. B* **2005**, *109*, 6366–6374.

(4) Moura, L.; Mishra, M.; Bernales, V.; Fuentealba, P.; Padua, A. A.; Santini, C. C.; Costa Gomes, M. F. *J. Phys. Chem. B* **2013**, *117*, 7416–7425.

(5) Xing, H.; Zhao, X.; Li, R.; Yang, Q.; Su, B.; Bao, Z.; Yang, Y.; Ren, Q. *ACS Sus. Chem. Eng.* **2013**, *1*, 1357–1363.

(6) Anthony, J. L.; Anderson, J. L.; Maginn, E. J.; Brennecke, J. F. *Chem. Eng. Process. Process Intensif.* **2010**, *49*, 192–198.

(7) Zhang, J.; Zhang, Q.; Qiao, B.; Deng, Y. *J. Chem. Eng. Data* **2007**, *52*, 2277–2283.
